# Resequencing of 1,143 *indica* rice accessions reveals important genetic variations and different heterosis patterns

**DOI:** 10.1038/s41467-020-18608-0

**Published:** 2020-09-22

**Authors:** Qiming Lv, Weiguo Li, Zhizhong Sun, Ning Ouyang, Xin Jing, Qiang He, Jun Wu, Jiakui Zheng, Jiatuan Zheng, Shaoqing Tang, Renshan Zhu, Yan Tian, Meijuan Duan, Yanning Tan, Dong Yu, Xiabing Sheng, Xuewu Sun, Gaofeng Jia, Hongzhen Gao, Qin Zeng, Yufei Li, Li Tang, Qiusheng Xu, Bingran Zhao, Zhiyuan Huang, Hongfeng Lu, Na Li, Jian Zhao, Lihuang Zhu, Dong Li, Longping Yuan, Dingyang Yuan

**Affiliations:** 1 State Key Laboratory of Hybrid Rice, Hunan Hybrid Rice Research Center, Hunan Academy of Agricultural Sciences, 410125 Changsha, China; 2 Longping Branch of Graduate School, Hunan University, 410125 Changsha, China; 3 Huazhi Biotech Co. Ltd, 410125 Changsha, China; 4 Novogene Bioinformatics Institute, 100089 Beijing, China; 5 Key Laboratory of Southwest Rice Biology and Genetic Breeding, Ministry of Agriculture, Rice and Sorghum Research Institute, Sichuan Academy of Agricultural Sciences, 618000 Deyang, China; 6 Rice Research Institute, Fujian Academy of Agricultural Sciences, 350019 Fuzhou, China; 7 State Key Laboratory of Rice Biology, China National Center for Rice Improvement, China National Rice Research Institute, 310006 Hangzhou, China; 8 State Key Laboratory of Hybrid Rice, College of Life Sciences, Wuhan University, 430072 Wuhan, China; 9 College of Agronomy, Hunan Agricultural University, 410128 Changsha, China; 10 State Key Laboratory of Plant Genomics, Institute of Genetics and Developmental Biology, Chinese Academy of Sciences, Beijing, 100101 China; 11 Southern Regional Collaborative Innovation Center for Grain and Oil Crops in China, College of Agronomy, Hunan Agricultural University, 410128 Changsha, China

**Keywords:** Agricultural genetics, Agricultural genetics, Comparative genomics, Plant hybridization

## Abstract

Obtaining genetic variation information from *indica* rice hybrid parents and identification of loci associated with heterosis are important for hybrid rice breeding. Here, we resequence 1,143 *indica* accessions mostly selected from the parents of superior hybrid rice cultivars of China, identify genetic variations, and perform kinship analysis. We find different hybrid rice crossing patterns between 3- and 2-line superior hybrid lines. By calculating frequencies of parental variation differences (FPVDs), a more direct approach for studying rice heterosis, we identify loci that are linked to heterosis, which include 98 in superior 3-line hybrids and 36 in superior 2-line hybrids. As a proof of concept, we find two accessions harboring a deletion in *OsNramp5*, a previously reported gene functioning in cadmium absorption, which can be used to mitigate rice grain cadmium levels through hybrid breeding. Resource of *indica* rice genetic variation reported in this study will be valuable to geneticists and breeders.

## Abstract (web summary)

Hybrid rice cultivars are widely planted around the world. Here, the authors resequence 1,143 indica accessions, focusing on the parents of superior hybrid rice lines in China, and reveal genetic loci that are associated with heterosis via measuring frequency of parental variation difference (FPVD).

## Introduction

*Indica* (xian) rice, one of the two major subspecies of Asian cultivated rice (*Oryza sativa*)^1^, is a staple food for people in many Asian countries. According to a previous study^2^, *indica* rice can be divided into two major genetic subgroups: *indica I* (*IndI*) and *indica II* (*IndII*). Heterosis is the phenomenon in which the first filial generation of two parent lines outperforms its homozygous parents. Taking advantage of heterosis, the commercial breeding of hybrid rice, which started in China in the 1970s and has spread to the other main rice-producing countries in Asia, has greatly secured the food supply in these countries^3^. Since then, many important *indica* hybrid parents have been developed. Although there are *indica-japonica* and *japonica-japonica* hybrids, the most common rice hybrids are *indica-indica* hybrids, or *indica* hybrids for short.

There are two types of *indica* hybrid rice: 3-line hybrids and 2-line hybrids. The former, which have been commercially available since the 1970s, are generated from crosses between a cytoplasmic male sterile (CMS) line with a sterility gene in the mitochondrial genome and a nonfunctional fertility gene in the nuclear genome, a restorer line (3R) with a functional fertility gene in the nuclear genome that can reverse sterility, and a maintainer line (3M). The 2-line hybrids, which have been commercially available since the 1990s, are generated from a bifunctional line that can behave either as a genic male sterile (GMS) line or a normal line that can reproduce itself (depending upon environmental conditions such as day length and temperature) and a restorer line, which can be either a 2-line restorer (2R) or a 3-line restorer (3R)^4–8^. It has been thought that a small number of different genes are likely responsible for 3- and 2-line yield heterosis, as based on studies on some *indica* hybrids^9,10^.

The first large rice resequencing project, the 3000 Rice Genomes Project in 2014, included many *indica* rice varieties, but only 322 of them were from China^11^. Despite other rice resequencing studies, the read lengths were short (between 73 and 90 bp), and the sequencing depth was low (between one- and threefold)^2,12^. Many important Chinese *indica* rice accessions, such as Peiai64 (an important germplasm), Quan9311A (a popular CMS), Mianhui146 (a popular 3R), and Shen08S (a popular GMS), were not included in these studies.

To date, almost all rice genomic studies use the *japonica* Nipponbare genome as the reference genome. However, as reported in the releasing of the genome of *indica* R498, it is different from that of Nipponbare in many ways^13^. Thus, it would be more accurate by aligning *indica* genetic variation reads to the R498 genome rather than the *japonica* Nipponbare genome. In addition, increasing read length and coverage depth can help to capture accurate and complete genetic variation information for the *indica* accessions.

In the present study, we select 1143 *indica* accessions, mostly comprising the major *indica* accessions used in rice breeding and production in the last 50 years in three different *indica*-growing environments in China (the upper reaches of the Yangtze River, the middle and lower reaches of the Yangtze River, and southern China), with a particular focus on the parents of superior *indica* hybrid rice lines. Most of these important accessions have never been resequenced. We identify their genetic variations, using the R498 genome as the reference genome, perform the kinship analysis, and find different hybrid rice crossing patterns in 3- and 2-line superior hybrid lines. Because most-shared parental genetic differences among superior hybrids should be the most relevant to heterosis, we also identify the most-shared single-nucleotide polymorphism (SNP) and indel differences between two parents among all superior hybrids for each type of 3- and 2-line systems, and further identify the different loci associated with heterosis in 3- and 2-line hybrids. We further detect a natural mutation in two accessions that can be used to mitigate cadmium contamination in rice grains.

## Results

### Resequencing

As detailed in Supplementary Data 1, the 1143 *indica* accessions in this study included 211 CMS, 110 GMS, 294 3R, and 81 2R accessions (696 in total), representing the parents of the majority of *indica* hybrid rice accessions that have been widely planted in southern China for almost the last 50 years; some accessions grown internationally were also included. There were also 15 3M, 296 conventional rice (CR), and 136 germplasm rice (GR) accessions that have been used to improve hybrid rice parents and CR accessions. Of the total accessions, 136 were obtained from the International Rice Research Institute and countries other than China (most of which were GM or CR accessions), and the remainder were obtained from China. The raw next-generation sequencing dataset is approximately 3.8 TB and includes 54.7 billion paired-end reads. The average read size is 150 bp (much longer than 87 bp, the average read length for the 3000 Rice Genomes accessions^11^).

### Identification of SNPs and indels

Except for recently published MBKbase^14^, all major genetic studies on rice to date have used the genome of Nipponbare, a *japonica* rice (the other major subspecies of cultured Asian rice), as the reference genome^1,9,11,15–18^. Because the genome of *indica* rice R498, which was constructed using recent technologies, is more complete (17 Mb longer) and continuous than that of Nipponbare^13^, and is the same subspecies as the accessions we studied, we chose the R498 nuclear, mitochondrial, and chloroplast genomes as the reference genomes for this study.

The average mapping rate of the sequencing reads to the R498 nuclear genome was approximately 99.1%. The average genome coverage depth was approximately 17.3× (Supplementary Data 2), slightly greater than the 14× for the average depth for the 3000 Rice Genomes accessions^11^. SNP and indel identification using the R498 nuclear, mitochondrial, and chloroplast genomes yielded a total of 19.3 million raw SNPs and 2.8 million indels across 1143 accessions, after initial SNP calling. A total of 3.86 million high-quality nuclear SNPs were obtained after filtering with a minor allele frequency greater than 0.01 (Supplementary Table 1 and Methods), and a total of 0.717 million high-quality indels were obtained after filtering (Supplementary Table 2). The nonsynonymous/synonymous substitution ratio for the nuclear SNPs in the CDS regions was 1.55, similar to 1.59 found for *indica* Guangluai-4 (ref. ^19^), and slightly higher than the value of 1.46 in the 3000 Rice Genomes Project^11^, indicating stronger positive selection in *indica* rice. A total of 452 and 102 high-quality SNPs for the mitochondrial and chloroplast genomes, respectively, were also obtained. The average SNP densities in the nuclear, mitochondrial, and chloroplast genomes were approximately 9.9, 0.86, and 0.76 SNPs/kb, respectively, indicating that the mitochondrial and chloroplast genomes are more conserved than the nuclear genome, which is consistent with previous reports^20,21^.

### Phylogenetic and kinship analyses

First, we performed phylogenetic analysis using the nuclear SNP data and found that most of the CMS, GMS, and GR accessions are grouped into separate clades (Supplementary Fig. 1). Moreover, most 3R and 2R accessions grouped together. However, our results did not show any clear separation between the three *indica*-growing environments or the different time periods. This likely reflects the history of the development of Chinese *indica* rice, in which elite accessions were widely shared among rice breeders nationwide and then further modified for improvement in different environments^4,5^. We also performed phylogenetic analysis using 452 high-quality SNPs from the mitochondrial genome. Most CMS accessions clearly grouped together (Supplementary Fig. 2), as expected from the important role that the mitochondrial genome plays in male sterility in CMS accessions.

In addition to phylogenetic analysis, we performed linkage disequilibrium (LD) analysis. The results showed that the CR accessions had the fastest rate of decline, indicating the highest diversity for this group (Supplementary Fig. 3). The GMS and 2R accessions exhibited the slowest rates of decline, indicating the least diversity, probably because the 2-line hybrid system was developed 20 years later than the 3-line hybrid system; thus, there have been fewer parents in the 2-line system. The rates of LD decline are similar to the rates in previous reports^15,16^.

**Fig. 1:**
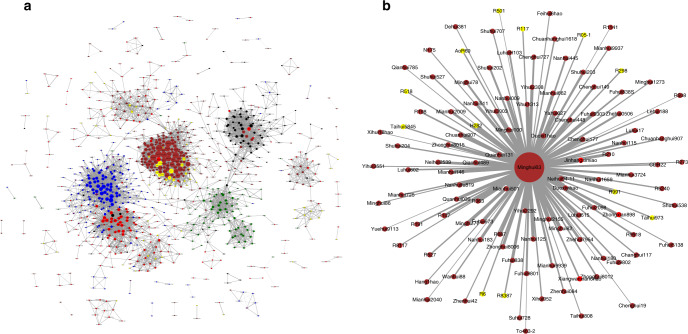
Kinship relationships among accessions. **a** All kinship relationships with kinship coefficients greater than 0.45. Of 1143 accessions, 992 formed 50 multiple-member clusters and 7376 relationships. The remaining 151 accessions did not have any relationship above the cut-off value and thus are not shown. The largest cluster contains 772 accessions and several subclusters. **b** Minghui63 and 113 related accessions with kinship coefficients greater than 0.45. In both **a** and **b**: circle sizes represent the degree of relationship, i.e., the number of lines connected to each circle; line widths are based on the coefficients between two accessions; circles are in red for conventional rice (CR), yellow for 2-line restorers (2R), green for 2-line photoperiod genic and thermosensitive male sterile (GMS) lines, blue for 3-line cytoplasmic male sterile (CMS) lines, brown for 3-line restorers (3R), purple for 3-line maintainers (3M), and black for germplasm rice (GR). Source data are provided as a Source Data file.

Because not all accessions that were used to develop these 1143 *indica* accessions were resequenced and complicated crossbreeding is frequently involved in developing a new line, it is difficult to reconstruct the genealogy of these accessions from genetic variation data. Instead, we performed kinship analysis by calculating kinship coefficients for all pairwise comparisons among the 1143 *indica* accessions using GEMMA^22^ (v0.98.1-0). We chose a kinship coefficient cut-off of 0.45 (10% below the first-degree value 0.5, to allow for some errors) and then plotted the relationship data with kinship coefficients greater than the cut-off value (Supplementary Data 3) using Cytoscape (http://www.cytoscape.org/). The results showed 992 accessions with kinship coefficients greater than 0.45, forming 50 multiple-member clusters and 7376 relationships (Fig. 1a and Supplementary Fig. 4). The remaining 151 accessions did not display a relationship with a kinship coefficient above the cut-off value. The largest cluster contained 772 accessions, most of which were further divided into eight groups: a restorer group, a CMS group, two GMS groups, two CR groups, and two mixed groups. We found that Minghui63 has close relationships with 98 3R, 12 2R, and 3 CR accessions (Fig. 1b and Supplementary Fig. 5). Zhenshan97A, an important CMS accession developed in the 1970s and widely used and shared since, has close relationships with 68 other accessions (Supplementary Fig. 4 and Supplementary Data 3). These close yet complicated relationships also reflect the complicated rice breeding history in China.

### Population structure and superior hybrid crossing patterns

**Fig. 2:**
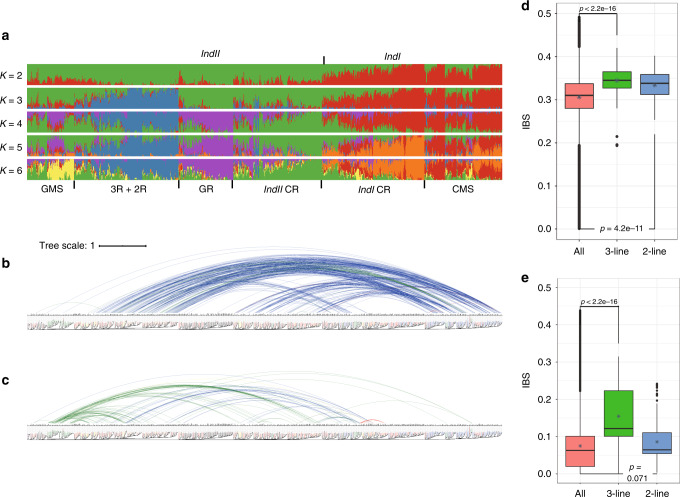
Genetic analysis of superior indica hybrids. **a** Population structure analysis of 1143 *indica* accessions. The results for *K* values from 2 to 6 are shown. When *K* = 2, two genetic groups match the *IndII* and *IndI* subgroups (see “Methods”). When *K* = 6, the six genetic groups generally correspond to GMS, restorer (3R + 2R), GR, *IndII* CR, *IndI* CR, and CMS lines, with some exceptions in each group. **b**, **c** Crossing patterns of the superior 3- (**b**) and 2-line (**c**) hybrids. The top curves connect two parents of superior hybrids: blue: *IndII* restorers crossed with *IndI* male sterile lines; green: *IndII* restorers crossed with *IndII* male sterile lines; red: *IndI* restorers crossed with *IndI* male sterile lines; yellow: *IndII* restorers crossed with *IndII* male sterile lines. A nuclear-genome phylogenetic tree in the horizontal format is shown below the curves in the same color schema as in Fig. 1. **d** Average nuclear-genome genetic distances between two parents for all 1143 accessions (*n* = 652,653) and superior 3- (*n* = 415) and 2-line (*n* = 136) hybrids (all: min = 0.001, max = 0.491; 3-line: min = 0.194, max = 0.420; 2-line: min = 0.253, max = 0.402). **e** Average mitochondrial genome genetic distances between two parents for all 1143 accessions (*n* = 652,653) and superior 3- (*n* = 415) and 2-line (*n* = 136) hybrids (all: min = 0.000, max = 0.438; 3-line: min = 0.001, max = 0.315; 2-line: min = 0.003, max = 0.242). In **d**, **e**, the *p* values by two-tailed unpaired Welch’s *t-*tests are shown on diagrams. Data representation: the middle line: median; the asterisk: mean; the lower and upper hinges: the first and third quartiles; the upper whisker extends from the hinge to the largest value no further than 1.5 × IQR (inter-quartile range) from the hinge. The lower whisker extends from the hinge to the smallest value at most 1.5 × IQR of the hinge. Data beyond the end of the whiskers, which were considered as the outlying points, are plotted individually. Source data underlying figures **b**–**e** are provided as a Source Data file.

We analyzed the population structure of the 1143 *indica* accessions using ADMIXTURE^23^. According to the results (Fig. 2a, *K* = 2), these accessions can be divided into two genetic groups that closely match the two *indica* subgroups (*IndI* and *IndII*) defined by Xie et al.^2^, which were determined by comparing to Zhenshan97, a typical *IndI* accession, and Minghui63, a typical *IndII* accession (see “Methods”). Among the 1143 accessions, 393 were classified as *IndI*, and the remaining 750 as *IndII*. Further analysis of the 211 CMS accessions showed most (186) to be *IndI*; most (88) of the 110 GMS accessions and most of the 3R, 2R, and GR accessions are *IndII* (Supplementary Table 3). When we increased *K* to 6, all accessions were divided into six genetic groups that generally corresponded to GMS lines, restorers (3R + 2R), GR, *IndII* CR, *IndI* CR, and CMS lines (Fig. 2a, *K* = 6), with some exceptions in each group.

We also examined parental crossing patterns for each of the superior 3- and 2-line hybrid rice cultivars (Supplementary Data 4). Among 415 superior 3-line hybrids, 382 were found to be crosses between *IndII* restorers and *IndI* CMS lines. Among 136 2-line hybrids, 95 are crosses between *IndII* restorers and *IndII* GMS lines, and 30 are crosses between *IndII* restorers and *IndI* GMS lines (Fig. 2b, c, Supplementary Figs. 6 and 7, and Supplementary Table 4). For 3- or 2-line hybrid rice, crosses using *IndI* restorers are rare. The superior 3- and 2-line crossing patterns appear to differ; however, the superior 3-line hybrids are more limited to crosses between certain *IndII* accessions as restorers and certain *IndI* accessions as CMS lines. Although we identified a few crosses between two parents that are very closely related to each other in both the 3- and 2-line superior hybrids, most crosses were detected as being between two parents in two different or distant clades in the nuclear-genome phylogenetic tree.

### Heterosis and genetic distances

Next, we calculated nuclear and mitochondrial genome genetic distances between the two parents of each of the superior *indica* hybrids in both 3- and 2-line systems. For the nuclear genome, the average genetic distance between the parents for each of the superior 3- and 2-line hybrids (0.345 and 0.334, respectively) was indeed greater than that for all possible combinations of all 1143 accessions (0.306; Fig. 2d). Regarding the mitochondrial genome, the average genetic distance between the parents for each of the superior 3-line hybrids (0.155) was markedly greater than that for all possible combinations of 1143 accessions (0.075). In contrast, the average genetic distance between the parents for each of the superior 2-line hybrid rice lines (0.086) was very similar to that for all possible combinations (Fig. 2e). Although the hybrids only carry maternal mitochondria, these results confirm that the difference in mitochondrial genomes between the two parents for 3-line hybrid rice is important, and that the parents of 2-line hybrid rice do not require such a difference (as the sterility and fertility of GMS lines are both controlled by the nuclear genome).

### Identification of loci associated with heterosis

Finding loci involved in rice heterosis is important for improving rice yield. In the last several years, two rice heterosis studies have taken advantage of accurate and cost-effective next-generation sequencing technology. By analyzing computationally delineated parent information from a fixation index (*F*_st_) analysis without separating the 3- and 2-line systems from each other, Huang et al.^17^ identified 31 highly differentiated loci with a total length of 22.3 Mb between two parental populations. In their new study, Huang et al.^9^ used another gene identification method—resequencing and genetically mapping very large *F*_2_ populations of 17 hybrid rice cultivars at just 0.2× genome coverage —and showed that 3- and 2-line hybrids had a small number of different genes that likely contributed to yield heterosis. Here, the abundance of genetic variation data for many important parents of superior rice hybrids allowed us to take a more direct approach by focusing on the parental genetic differences of superior hybrids. Because our study revealed that superior 3- and 2-line hybrids have different crossing patterns (Fig. 2b, c) and earlier studies also indicated that different genes were involved in the superior 3- and 2-line hybrid rice cultivars^9,17^, we analyzed the two hybrid systems separately.

**Fig. 3:**
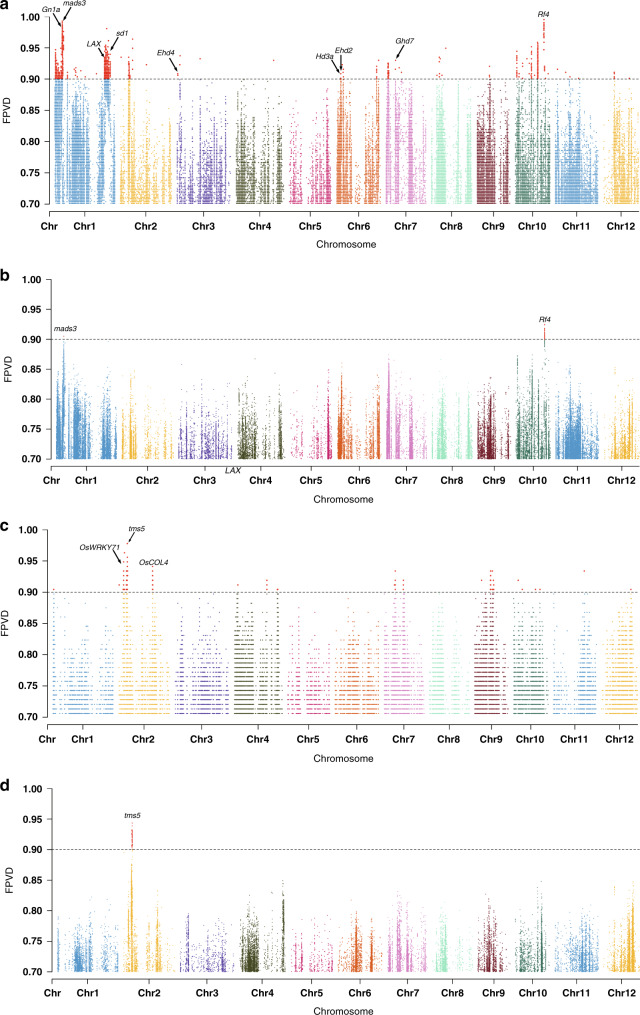
Genome-wide SNP FPVD analyses. **a** Superior 3-line hybrids (*n* = 415). **b** Simulated inferior 3-line hybrids (*n* = 61,619). **c** Superior 2-line hybrids (*n* = 136). **d** Simulated inferior 2-line hybrids (*n* = 51,114). The inferior 3-line hybrids are the results of simulated crosses between 294 3R and 211 CMS accessions for the 3-line system, with the real superior 3-line hybrids excluded. The inferior 2-line hybrids are the results of simulated crosses between 275 3R + 2R restorers and 110 GMSs, with the real superior 2-line hybrids excluded. Some agronomically important genes located in the loci that we identified are labeled. Source data are provided as a Source Data file.

For each of the 415 superior 3-line hybrids, we first compared the variations for each of the 3.86 million high-quality SNPs between two parents. Next, for each SNP position, we obtained the total of hybrids with different parental variations and then calculated the frequencies of parental variation differences (FPVDs) for each SNP in all 415 hybrids and plotted the FPVD values for all SNP positions (Fig. 3a). We did not detect any SNPs or indels with an FPVD of 1 (100%) but did notice that the FPVD values of some SNPs were much higher than those of other SNPs. To help determine an appropriate cut-off value to identify high-FPVD SNPs that are likely associated with heterosis, we simulated inferior hybrids for the 3-line system because either the real inferior hybrids were not well documented by the rice breeders or such information was not available. We used all possible hybrids between the 294 3R and 211 CMS accessions, with the real superior hybrids excluded, as the inferior hybrid set, and then performed FPVD analysis on this set (Fig. 3b). The two highest peaks in the FPVD plot for the 3-line inferior hybrids span two important genes: *mads3*, a gene on chromosome 1 that regulates late anther development and pollen formation^24^, and *Rf4*, a gene on chromosome 10 that restores fertility for WA-type CMS lines^25^ (Supplementary Table 5). We performed the same analysis on indels for the superior hybrid set and the indel FPVD results were very similar to those of SNPs (Supplementary Fig. 8a).

As the peak SNP FPVD values located in both the *mads3* and *Rf4* gene regions were above 0.9, we chose this value as the FPVD cut-off for a superior hybrid FPVD analysis. Using this cut-off value, we found 98 loci that span 3218 SNPs and 539 indels, with a minimum locus length of 100 kb, a maximum length of 11.6 Mb, and a total length of 18.5 Mb (Supplementary Fig. 9a and Supplementary Data 5). These highly differentiated loci are mostly located on chromosomes 1, 2, 6, 7, and 10, with none on chromosome 5. In addition to *mads3* and *Rf4*, these loci span several known genes that control agronomically important traits, such as heading control (*hd3a*^26^, *Ehd2* (ref. ^27^) (both were also found by Huang et al.^17^), and *Ehd4* (ref. ^28^), pleiotropy (*Ghd7* (ref. ^29^), grain number (*Gn1a*^30^), panicle initiation (*LAX1* (ref. ^31^), and dwarfism (*Sd1* (ref. ^32^). We also mapped 31 highly differentiated loci discovered by Huang et al.^17^ to the R498 reference genome and compared them with the loci we found. Seventeen of the 31 loci from Huang’s study partially overlap with 29 of the 98 loci identified in our study, and most overlaps are on chromosomes 1, 2, and 7 (Supplementary Fig. 9a). It is possible that not all 98 loci found in this study contribute to heterosis, and that some loci may be an artifact of the background differences between the parental populations. Nonetheless, the FPVD results from the simulated inferior hybrids indicate that background differences are unlikely to have caused false positives among the 98 loci we identified.

For the 136 superior 2-line hybrids, we performed the same analysis as for the 3-line hybrids. Using the 0.9 FPVD cut-off for the FPVD analysis of the superior 2-line hybrid, we identified 36 loci that span 472 SNPs and 108 indels, with a minimum locus length of 100 kb, a maximum length of 800 kb, and a total length of 5.4 Mb (Fig. 3c, Supplementary Fig. 9b, and Supplementary Data 6). Unlike those in the 3-line rice hybrids, these loci are mainly located on chromosomes 2, 4, 7, and 9, and none are located on chromosomes 3, 5, and 6. In addition to *tms5*, these loci span several known genes that control important agronomic traits, such as *OsCOL4* for flowering time^33^ and *OsWRKY71* (ref. ^34^) and *OsHPL2* (ref. ^35^) for bacterial blight resistance. Indel FPVD analysis showed a very similar pattern (Supplementary Fig. 8b). We also compared the 31 loci found by Huang et al.^17^ with the loci we found in the 2-line hybrids. However, unlike those of the 3-line hybrids, only 2 of Huang’s 31 loci partially overlapped with 3 of the 36 loci identified in this study (Supplementary Fig. 9b). For the inferior hybrid set for the 2-line system, we used all possible hybrids between all 375 restorers, i.e., both 2R and 3R accessions, and all 110 GMS accessions, with the real superior 2-line hybrids excluded; we then performed FPVD analysis on the inferior hybrids. The highest peak in the SNP FPVD plot for the 2-line inferior hybrids spans *tms5*, which is responsible for thermosensitive male sterility in the 2-line system^36^, on chromosome 2 (Fig. 3d and Supplementary Data 7).

To further validate our FPVD results, we performed *F*_st_ analysis to compare the parental populations for each of the 3- and 2-line hybrid rice lines. For the 3-line hybrids, there were 380 genes, including *Rf4* and *mads3*, in the selective-sweep regions, mainly located on chromosomes 1, 6, 7, and 10 and with peaks similar to those in our 3-line FPVD study (Supplementary Fig. 10a and Supplementary Data 8). For the 2-line system, there were 359 genes, including *tms5*, in the selective-sweep regions, which were mainly located on chromosomes 2, 6, and 9 and had peaks at similar locations on chromosomes 2 and 9 as those in our 2-line FPVD study (Supplementary Fig. 10b and Supplementary Data 9). Although our *F*_st_ analysis compares two parental populations rather than two specific parents for a superior hybrid, the results largely confirmed our FPVD peak regions for both the superior 3- and 2-line hybrids.

### Gene PAV analysis and *OsNramp5* deletion mutants

**Fig. 4:**
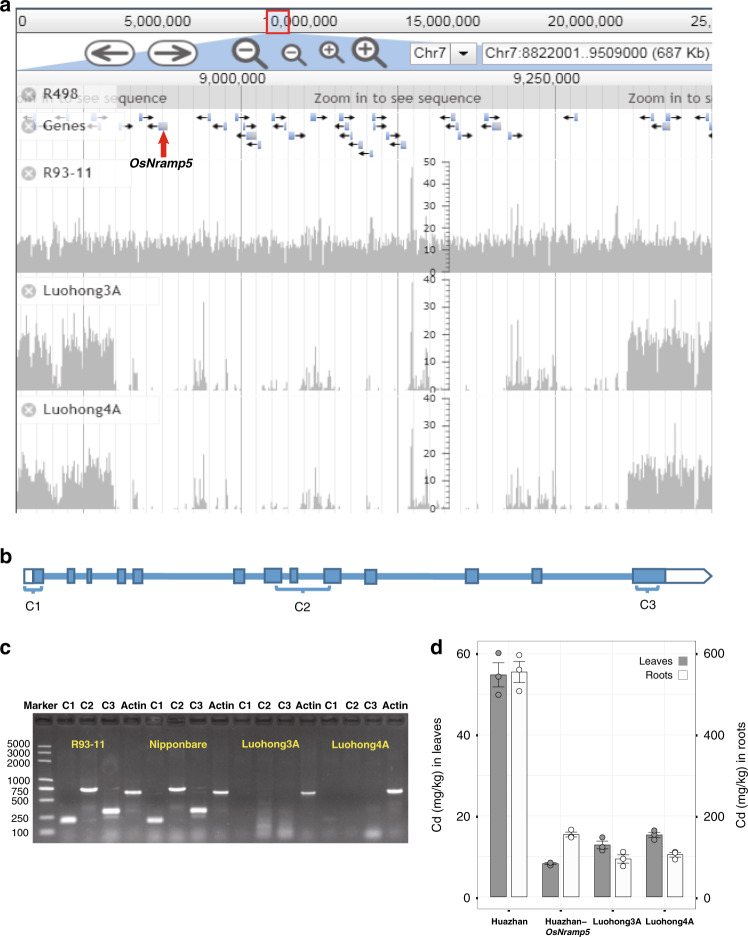
Genetic variation in *OsNramp5* in Luohong3A and Luohong4A. **a** Deletion that spans *OsNramp5* (marked by the red arrow) in Luohong3A and Luohong4A shown using JBrowse^52^ (v1.12.3). R93-11, a commonly used 2R, was used as a control to demonstrate no large deletion. **b** Gene structure of *OsNramp5* in Nipponbare showing the location of regions used for PCR: C1 on exon 1, C2 on exons 7 and 9, and C3 on exon 13. **c** Agarose gel electrophoresis of PCR products for accessions R93-11 (positive control), Nipponbare (positive control), Luohong3A, and Luohong4A using three pairs of primers targeting the C1, C2, and C3 regions shown in **b**. The rice actin gene served as the PCR DNA control. The marker sizes (bp) are shown on the left side. Three biological replicates were analyzed for each sample and a representative gel image is shown. **d** Bar plot for Cd content in leaves (dark) and roots (white) of Huazhan (positive control), Huazhan-*OsNramp5* (*OsNramp5* knockout^39^, negative control), Luohong3A, and Luohong4A. Mean values ± s.d. (*n* = 3) are shown. All individual data points are shown as dots. Source data underlying **c** and **d** are provided as a Source Data file.

We further performed gene presence/absence variation (PAV) analysis on 434 important loci and genes for all 1143 accessions, and found some genes to be completely absent from some accessions (Supplementary Data 10). One of these genes is *OsNramp5*, which controls cadmium (Cd) absorption^37^. Cd contamination in rice grains has become a problem in China and other countries in Asia, mainly due to water and soil contamination^38^. It has been reported that knockout of *OsNramp5* via CRISPR/Cas9 targeting exons 1, 7, and 9 or alteration of *OsNramp5* on exon 7 via ethyl methanesulfonate drastically decreases Cd accumulation in rice grains, roots, and shoots, with or without significantly lowering the yield^39–42^. Our gene PAV analysis results revealed a 408-kb deletion on chromosome 7 that spans the entire *OsNramp5* region in accessions Luohong3A and Luohong4A (Fig. 4a). To confirm the *OsNramp5* deletion, we performed PCR using three pairs of primers targeting exons 1, 7–9, and 13 of *OsNrapm5* (Fig. 4b), and the results confirmed the absence of this gene from these two accessions (Fig. 4c). Phenotyping experiments also verified that Cd contents in the leaves and roots of Luohong3A and Luohong4A were markedly lower than those in Huazhan (positive control) and were similar to those in Huazhan-*OsNramp5* plants, in which *OsNramp5* has been knocked out (Fig. 4d).

## Discussion

In this study, we used a variety of techniques to mine the vast genetic variation information for 1143 *indica* lines. Kinship analysis reveals the complicated many-to-many relationships that characterize the development of rice accessions. Our study also showed that superior 3- and 2-line hybrids have different crossing patterns. Furthermore, we employed a method called FPVD to identify the different loci in 3- and 2-line hybrids that are likely related to heterosis. Through the gene PAV study, we found two accessions with an important deletion that might be used to develop new accessions with low Cd accumulation.

The accession Colomlia links the CMS and GMS subclusters in kinship results, indicating that it was probably used to develop both CMS and GMS lines. Minghui63, a very common and widely used 3R accession that was initially developed in southern China in 1981, has also been widely shared among breeders and yielded hundreds of CR, 3R, and 2R accessions in all three *indica*-growing environments in China over the last 40 years (www.ricedata.cn), as supported by our kinship analysis results.

Our results showed that superior 3-line hybrids are more limited to crosses between certain *IndII* accessions as restorers and certain *IndI* accessions as CMS lines, likely due to the breeding history of how CMS and restorer lines were initially developed as well as the specific genetic interactions that are required between the mitochondrial and nuclear genomes in the 3-line system. The 2-line system is more flexible, as no interaction with the mitochondrial genome is required for sterility; thus, both 2R and 3R restorers can be used as males, though only some hybrids exhibit heterosis.

Because the sterility and fertility genes in simulated inferior hybrid sets for both 3- and 2-line hybrid systems were correctly identified using the FPVD analysis, we are confident that the FPVD method appropriately detected potential loci associated with heterosis. Although previous studies have used *F*_st_ to compare two parental populations and to identify loci associated with heterosis^17^, not all possible hybrids resulting from crosses between the two parental populations are superior; thus *F*_st_ analysis might miss loci that are truly associated with heterosis. Using very large *F*_2_ populations to identify loci associated with heterosis, as demonstrated in Huang et al.^9^, requires much resequencing and phenotyping and is thus very laborious and limited to studies on a small number of hybrids. Compared with these two previously published methods, the FPVD method should be simpler to perform and more accurate in detecting potential loci and genes associated with heterosis. Moreover, it should be applicable to other hybrid crops, such as maize, cotton, and pepper (*Capsicum*), as long as there are enough superior hybrids and the genetic variation information for their parents is known.

It was previously proposed that very few genes may be responsible for the yield heterosis of 3- and 2-line hybrids, but these findings were based on the limited number of superior hybrids^9^. Conversely our FPVD results are based on 551 superior hybrids and suggest that not only are different loci associated with heterosis in 3- and 2-line systems but that a greater number of loci and genes than previously expected are involved in rice heterosis. Theoretically, other than genes related to sterility and fertility, 3- and 2-line hybrid rice cultivars might share the same set of loci and genes associated with heterosis. The different loci and genes associated with heterosis between superior 3- and 2-line hybrids are probably a result of breeding history and differences in the flexibility of the two hybrid systems. It would be worth integrating the different loci associated with heterosis in the 3- and 2-line hybrids into the same lines to determine whether stronger heterosis can be achieved.

The natural deletion of *OsNramp5* found in our study might be valuable in commercial breeding to mitigate the Cd contamination problem in rice-producing countries, as many of these countries require regulatory procedures for gene-edited crops.

Given the large amount of genomic variation information relating to the parents of important hybrid rice lines, many more questions may be answered using this *indica* dataset, such as what the specific patterns of early, middle, and late hybrid rice crosses are in different environments. We will continue to mine these data to answer these questions. These large resequencing datasets for the 1143 important *indica* rice accessions and the analysis results, including SNP, indel, and gene PAV data, different crossing patterns, and different loci associated with heterosis in the 3- and 2-line *indica* hybrid rice, should be useful to both rice researchers and breeders and for both hybrid and CR improvement in the future.

## Methods

### Plant materials and growth conditions

The 1143 indica accessions in this study included 211 CMS, 110 GMS, 294 3R, 81 2R, 15 3M, 296 CR, and 136 GR accessions (Supplementary Data 1). Of the total, 136 accessions were obtained from the International Rice Research Institute and countries other than China (most of which were GM or CR accessions); the remaining accessions were obtained from China. Accessions were planted in a field in Changsha, Hunan, China.

### DNA sequencing and SNP calling

For each accession, a single individual was used for genome sequencing. Total DNA was extracted from the leaves of 1-month-old rice plants, and sequencing libraries with an approximately 300-bp insert size were prepared and sequenced using the Illumina NovaSeq 6000 platform by Novogene, Beijing, China. Reads containing adaptor sequences or stretches of ambiguous bases and those with low-quality scores were removed from the raw data. Paired-end reads were mapped to the R498 nuclear, mitochondrial, and chloroplast genomes^13^ with the Burrows-Wheeler Aligner^43^ (BWA, v0.7.8) using the command “BWA mem -t 4 -k 32 –M”. After BWA sorting, the “BWA rmdup” command was used to remove potential PCR duplicates, and only the pairs with the highest mapping quality were retained. After alignment, genomic variants (in genomic variant call format (GVCF) for each accession) were identified with the Haplotype Caller module and the GVCF model using Genome Analysis Toolkit (GATK) software^44^ (v3.8). All of the GVCF files were then merged into a single file. A widely accepted method of rice variation filtering (–minGQ 5–maf 0.01–max-missing 0.8–recode–recode-INFO-all) was used, yielding approximately 3.86 million high-quality SNPs and 0.717 million indels^1^. SNP/indel annotation was performed by mapping SNPs/indels from our dataset onto the gene structures defined by the R498 genome annotation using BEDtools^45^ (v2.26.0)^13^.

### Phylogenetic and linkage disequilibrium analyses

Pairwise identity-by-state (IBS) genetic distances for SNPs in the 1143 accessions were calculated using PLINK^46^ (v1.90). The nuclear SNP dataset of 3.86 million high-quality SNPs was employed for nuclear-genome-based phylogenetic analysis, and 452 high-quality mitochondrial SNPs were used for mitochondrial genome-based phylogenetic analysis. The average genetic distance of the entire 1143 accession population was the average of all pairwise distances between accessions. A neighbor-joining phylogenetic tree was then constructed using the R package APE^47^ (v4.1) with default parameters. Phylogenetic tree graphs were generated using iTOL^48^. For linkage disequilibrium (LD) analysis, PLINK with default parameters was employed to calculate complete and partial LD between each pair of SNPs. We moved 5000-SNP windows along each chromosome. In each window, the LD was estimated between pairs of marker loci plotted against the genetic distance. For every chromosome, we analyzed the values and significance of the squared correlation coefficient (*r*^2^) of any LD detected between polymorphic sites (*p* < 0.05).

### Kinship analysis

We calculated kinship coefficients for all pairwise comparisons among the 1143 *indica* accessions using GEMMA^22^ (v0.98.1-0) with default parameters and the same nuclear SNP dataset as in the phylogenetic analysis. We chose a cut-off kinship coefficient of 0.45, 10% below the theoretical first-degree value 0.5, to allow for some errors. Network images were generated using Cytoscape (v3.6.0, http://www.cytoscape.org/).

### Population structure and subgroup classification

The population structure of the 1143 accessions was determined using the ADMIXTURE^23^ (v1.3.0) program with default parameters and the same SNP dataset used in the above phylogenetic analysis. To study different genetic groups, we tested *K* values from 2 to 15 but only show results for *K* values of 2 to 6. Default settings were used in the analyses. Classification of our accessions into *IndI* and *IndII* groups was based on methods used in a previous study^2^ on two *indica* accessions: Zhenshan97, a typical *IndI* accession, and Minghui63, a typical *IndII* accession. Each accession was classified based on its two subpopulation components: accessions with values that indicated consistency with Zhenshan97 were classified as *IndI*; those with values that indicated consistency with Minghui63 were classified as *IndII*.

### Genetic distance calculations

To explore genetic distances between the parents of the superior *indica* hybrids, we calculated the nuclear and mitochondrial genome IBS genetic distances for these parents using PLINK^46^ (v1.90) with default parameters and high-quality SNP data and then calculated the average genetic distances for the superior 3- and 2-line hybrid rice lines. The average genetic distance for all possible combinations of all 1143 accessions was used as a control. The data were plotted using the box plot tool in R.

### Frequency of the parental variation difference analysis

The frequency of the parental variation difference (FPVD) for an SNP is calculated as follows:

1 $${\mathrm{FPVD}} = \mathop {\sum}\limits_{k = 1}^n {d_n/n}$$

where *n* is the total number of hybrids in a set and *d*_*n*_ is the value of the parent variation difference for the *n*th hybrid: if two parents have the same SNP variation at this SNP position, then *d*_*n*_ is 0; if they have different variations, then *d*_*n*_ is 1.

We calculated FPVDs for all high-quality SNP positions on all 12 chromosomes in a given hybrid set and then plotted all FPVDs for all SNP positions. We performed FPVD analysis for the superior 3- and 2-line hybrids separately. To construct inferior 3-line hybrids for comparison, we simulated all possible hybrids between 294 3R and 211 CMS accessions, excluded real superior 3-line hybrid rice lines, and used the remaining hybrids as inferior 3-line hybrids. Similarly, we simulated all possible hybrids from crosses between 375 3R + 2R restorer and 110 GMS accessions, excluded real superior 2-line hybrid rice lines, and used the remaining hybrids as inferior 2-line hybrids. For both 3- and 2-line systems, 0.9 was considered the FPVD cut-off. The boundaries for each locus were set as a 100 kb window around the position of an SNP with a 0.9+ FPVD value, and if two adjacent loci overlapped, they were merged into a longer locus. We used BLAST^49^ (v2.6.0) with parameters “-evalue 1e-200 -perc_identity 95 -max_target_seqs 1” to map 31 highly differentiated loci that were determined by Huang et al.^17^ to be present in the Nipponbare reference genome to the R498 genome, and the boundaries of some loci were extended somewhat during the mapping due to short insertions in the R498 genome. An indel FPVD analysis was performed in a similar way as for the SNP analysis described above.

### *F*_st_ analysis

The fixation index (*F*_st_) was estimated to assess the population differentiation of each pair of parents for the 3- and 2-line breeding systems. All genomic regions in 10-kb sliding windows were scanned in 10-kb steps, and regions containing SNPs within the top 1% of the distribution that presented higher differentiation than expected were defined as having stronger signals of selection within the selective-sweep regions.

### Gene PAV analysis

The read coverage of all genes in all 1143 samples was calculated using SAMTools depth^50^ (v1.7) with default parameters. The percentage of gene coverage was calculated using the number of bases in the gene with read coverage greater than 2 divided by the total length of the gene.

### PCR

Three pairs of primers, targeting exons 1 (C1), 7–9 (C2), and 13 (C3) of *OsNramp5* (Fig. 4b) in the Nipponbare reference genome IRGSP-1.0 (ref. ^51^) were used for PCR: C1: nrp5-C1f (5′-GTCACTACCACCATTCTCTTC-3′), nrp5-C1r (5′-CTTCATTAGCAGCTGATCATC-3′); C2: nrp5-C2f (5′-ATGCTGGTGTTCGTGATGGC-3′), nrp5-C2r (5′-AGGTGTCGAGGCTGAGGTTGG-3′); and C3: nrp5-C3f (5′-AGTGTTCTCGTGGTTCCTGGGTC-3′), nrp5-C3r (5′-GAGCGGGATGTCGGCCAGGTC-3′). Accessions R93-11 and Nipponbare served as positive PCR primer controls. The rice actin gene was used as a PCR DNA control. The standard PCR procedure was performed as follows: initial denaturation at 95 °C for 4 min, followed by 34 cycles of denaturation at 95 °C for 20 s, annealing at 57 °C for 20 s, and extension at 72 °C for 20 s, with additional extension for 5 min as the last cycle. The products were examined by 2% agarose gel electrophoresis. Huazhan-*OsNramp5*, an *OsNramp5* knockout line, was obtained from authors of a previous study^39^.

### Cd concentration measurement

One gram of rice tissue sample was dried at 70 °C for 2 days, ground into a powder, and digested with 25 ml of a 6:1 mixture of HNO_3_:HClO_4_ on an electric heating plate at 80 °C for 30 min, 150 °C for 30 min, and then 260 °C until there was no more evaporation. After cooling to room temperature, the residue was dissolved in 1% HNO_3_. The solution was diluted to 10 ml, and the Cd concentration was determined by inductively coupled plasma optical emission spectrometry (SPS3 ICP-720 OES; Agilent Technologies) at 226.502 nm.

### Reporting summary

Further information on research design is available in the Nature Research Reporting Summary linked to this article.

## Supplementary information

Supplementary information

Peer Review

Reporting Summary

Description of Additional Supplementary Files

Supplementary Dataset 1

Supplementary Dataset 2

Supplementary Dataset 3

Supplementary Dataset 4

Supplementary Dataset 5

Supplementary Dataset 6

Supplementary Dataset 7

Supplementary Dataset 8

Supplementary Dataset 9

Supplementary Dataset 10

## Data Availability

Data supporting the findings of this work are available within the paper and its Supplementary Information files. A reporting summary for this Article is available as a Supplementary Information file. The datasets generated and analyzed during the current study are available from the corresponding author upon request. All resequencing data have been deposited to NCBI with BioProject ID PRJNA656900. Source data are provided with this paper.

## Source data

Source Data
