# Supplementary material for: Resequencing of 1,143 indica rice accessions reveals important genetic variations and different heterosis patterns
Source: Nat Commun. 2020 Sep 22;11:4778. doi: 10.1038/s41467-020-18608-0 (PMC7508829; doi:10.1038/s41467-020-18608-0)
Supplement: Supplementary file 1 — Supplementary information [file 41467_2020_18608_MOESM1_ESM.pdf]

Resequencing of 1,143 *indica* rice accessions reveals important genetic variations and different heterosis patterns

Lv *et al.*

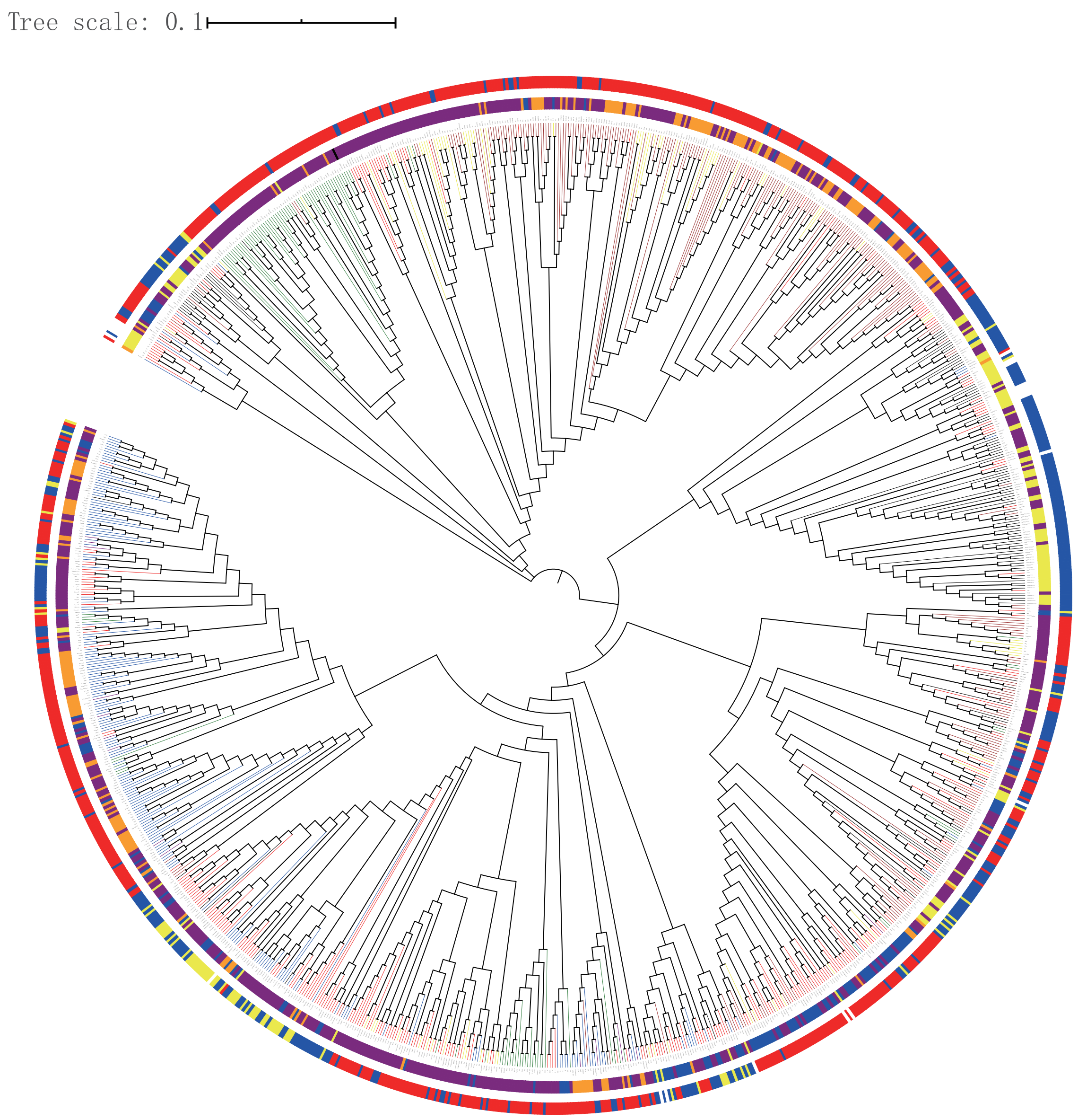

Supplementary Fig. 1 Phylogenetic analysis of 1,143 *indica* accessions based on nuclear SNP information. Center: phylogenetic tree and accession groups, with red for conventional rice (CR), yellow for 2-line restorers (2R), green for 2-line photoperiod genic and thermosensitive male sterile (GMS) lines, blue for 3-line cytoplasmic male sterile (CMS) lines, brown for 3-line restorers (3R), purple for 3-line maintainers (3M), and black for germplasm rice (GR). Inner circle: -growing environments, with orange for the upper reaches of the Yangtze River, purple for the middle and lower reaches, blue for southern China, and yellow for foreign countries. Outer circle: developmental periods, with yellow for before 1970, blue for 1971-1999, red for 2000-present, and white for unknown. Source data are provided as a Source Data file.

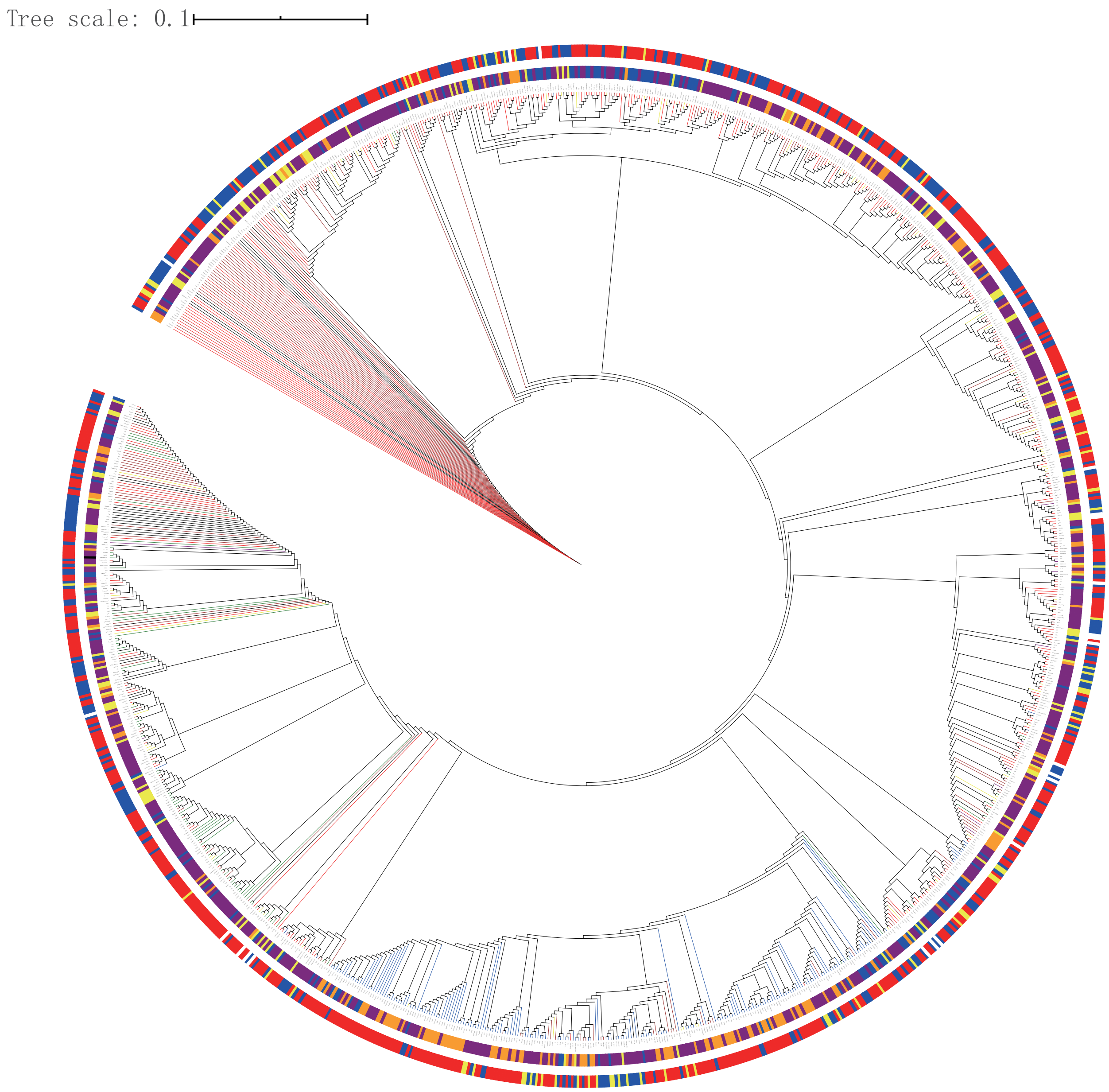

Supplementary Fig. 2 Phylogenetic analysis of 1,143 *indica* accessions based on mitochondrial SNP information. Center: phylogenetic tree and accession groups, with red for conventional rice (CR), yellow for 2-line restorers (2R), green for 2-line photoperiod genic and thermosensitive male sterile (GMS) lines, blue for 3-line cytoplasmic male sterile (CMS) lines, brown for 3-line restorers (3R), purple for 3-line maintainers (3M), and black for germplasm rice (GR). Inner circle: *indica*-growing environments, with orange for the upper reaches of the Yangtze River, purple for the middle and lower reaches, blue for southern China, and yellow for foreign countries. Outer circle: developmental periods, with yellow for before 1970, blue for 1971-1999, red for 2000-present, and white for unknown. Source data are provided as a Source Data file.

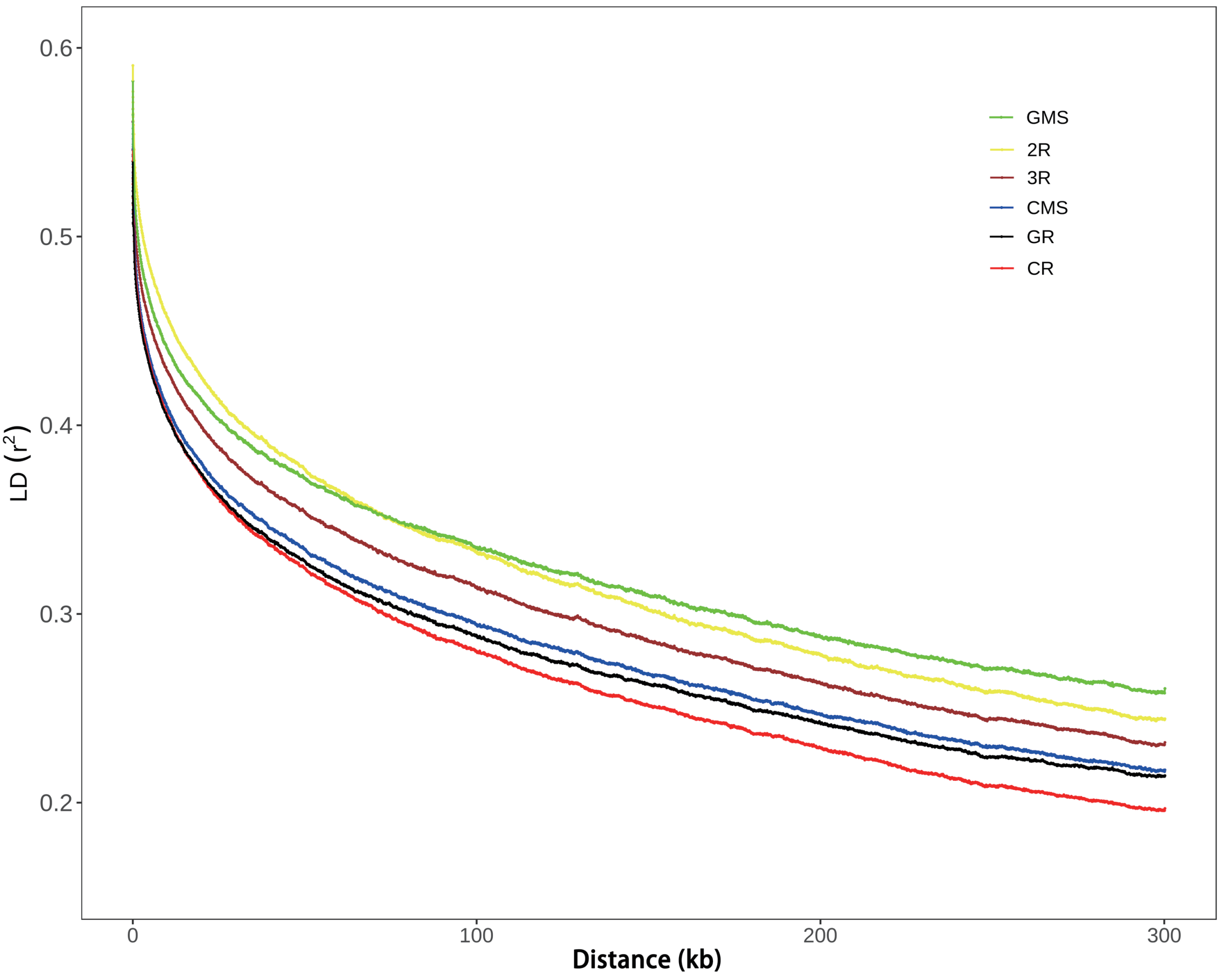

Supplementary Fig. 3 Linkage disequilibrium (LD) decay analysis for the 6 groups, expressed as r^2 as a function of inter-SNP distance for high quality SNPs. The 3-line maintainer (3M) group is not shown because of its small population size and its minimal effect on heterosis. Source data are provided as a Source Data file.

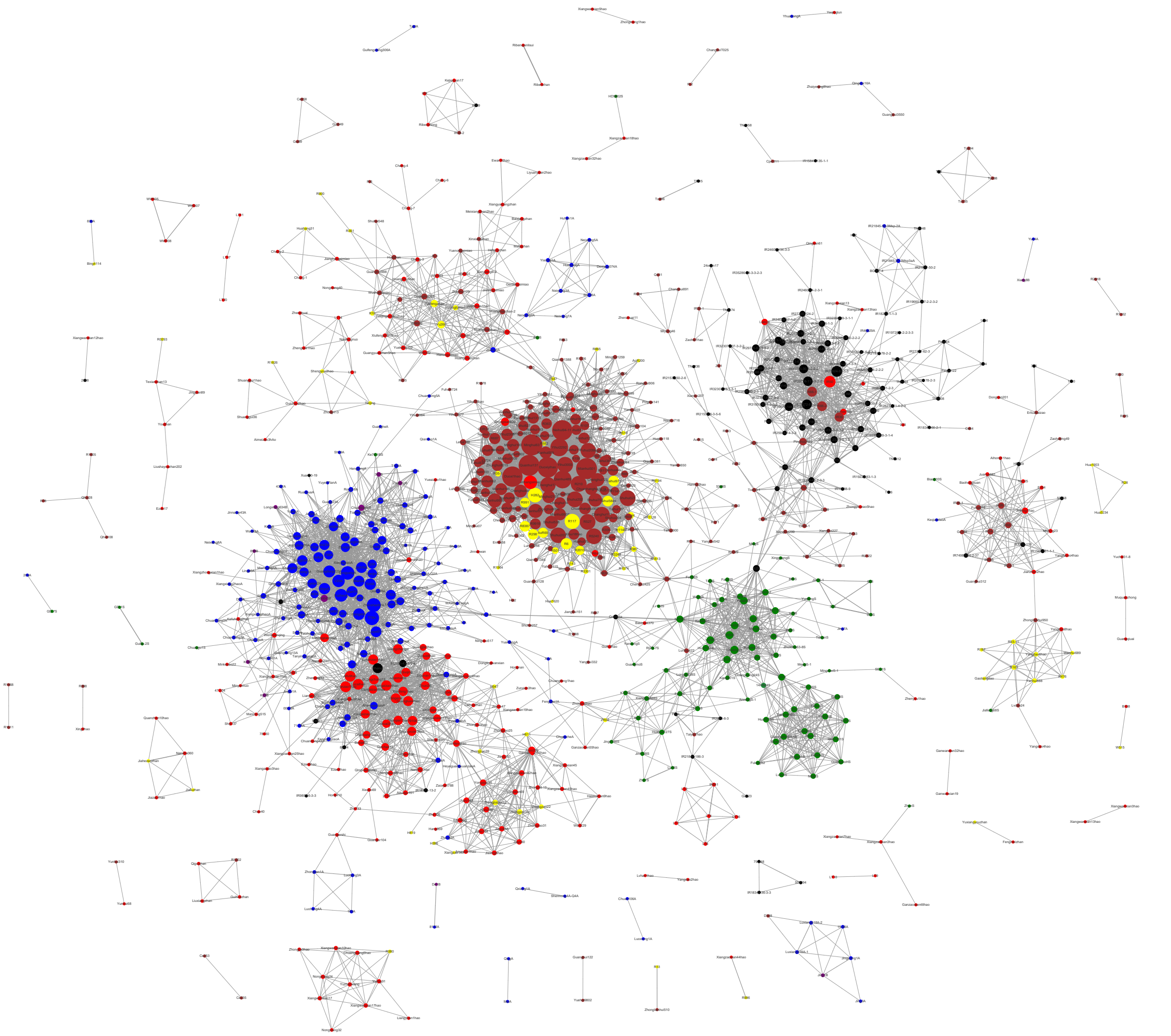

Supplementary Fig. 4 Kinship relationships among accessions with kinship coefficients greater than 0.45, in zoomable PDF format. Of 1,143 accessions, 992 formed 50 multiple-member clusters and 7,376 connections. The remaining 151 accessions did not have any relationship above the cut-off value and thus are not shown. The largest cluster contains 772 accessions and several subclusters. Circle sizes are mapped to the degree of relationship, i.e., the number of lines connected to that circle, and line widths are based on the coefficients between two accessions. Circles are in red for conventional rice (CR), yellow for 2-line restorers (2R), green for 2-line photoperiod genic and thermosensitive male sterile (GMS) lines, blue for 3-line cytoplasmic male sterile (CMS) lines, brown for 3-line restorers (3R), purple for 3-line maintainers (3M), and black for germplasm rice (GR). Source data are provided as a Source Data file.

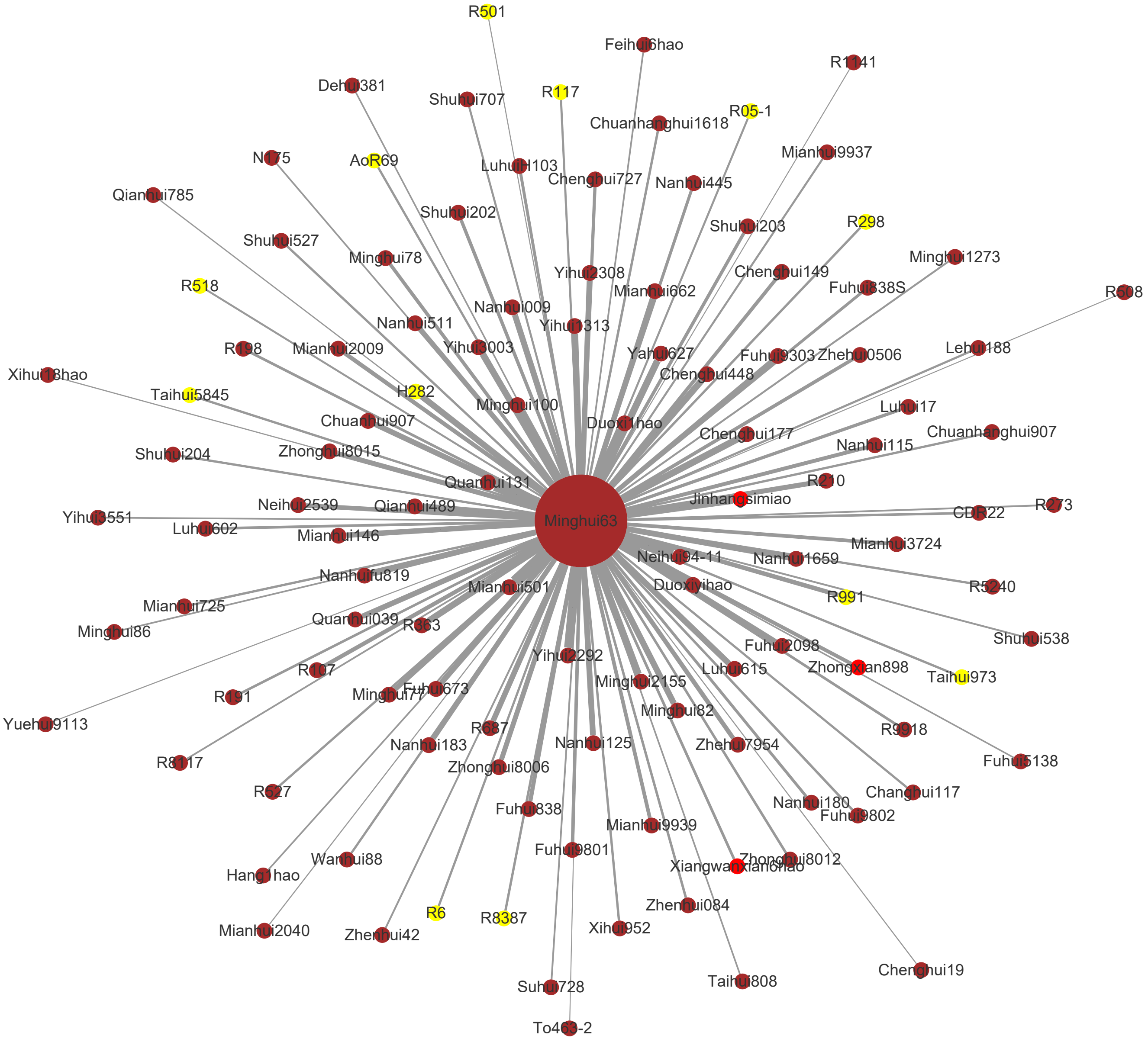

Supplementary Fig. 5 Relationships with Minghui63 with kinship coefficients greater than 0.45, in zoomable PDF format. Circle sizes are mapped to the degree of relationship, i.e., number of lines connected to that circle, and line widths are based on the coefficients between two accessions. Red indicates conventional rice (CR); yellow, 2-line restorers (2R); and brown, 3-line restorers (3R). Source data are provided as a Source Data file.

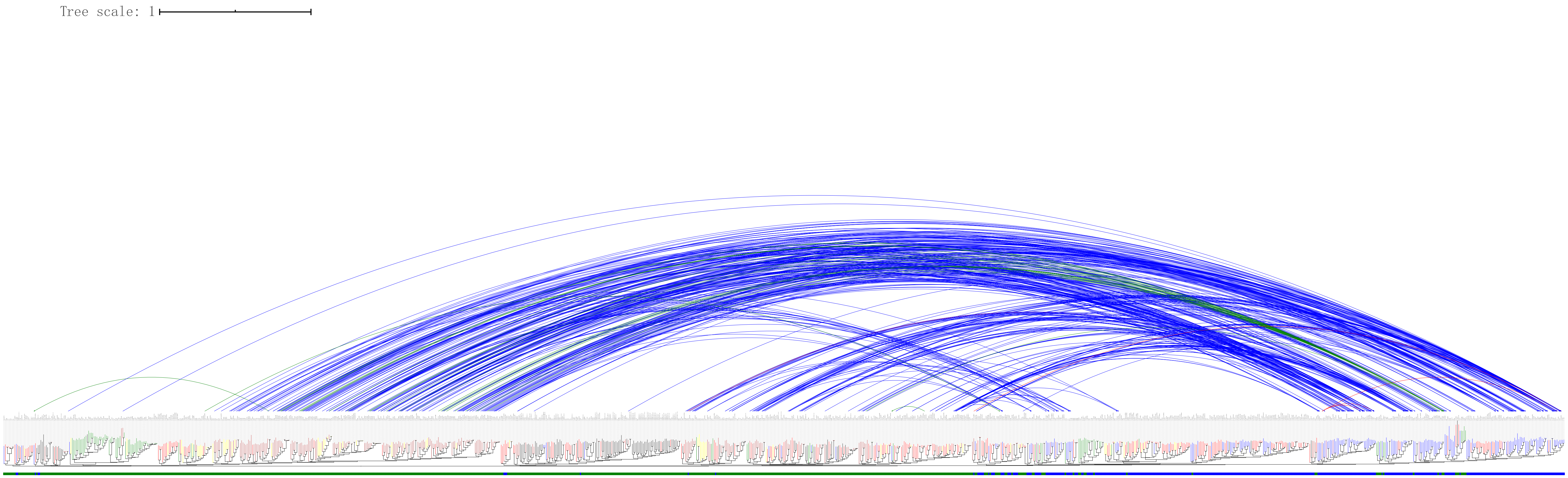

Supplementary Fig. 6 The crossing pattern of the superior 3-line hybrids, in zoomable PDF format. The top curves connect two parents of superior hybrids: blue, *IndII* restorers crossed with *IndI* male sterile lines; green, *IndII* restorers crossed with *IndII* male sterile lines; red, *IndI* restorers crossed with *IndI* male sterile lines; and yellow, *IndII* restorers crossed with *IndII* male sterile lines. The nuclear genome phylogenetic tree in horizontal format is shown below the curves in the same color schema as in Fig. 1. Source data are provided as a Source Data file.

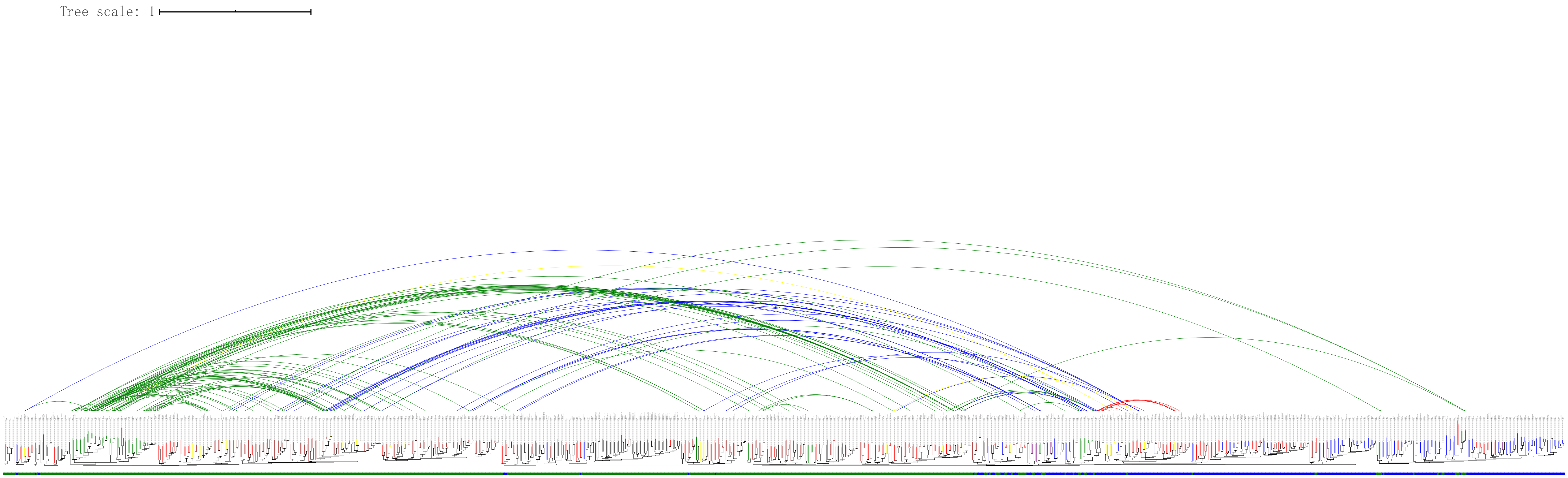

Supplementary Fig. 7 The crossing pattern of the superior 2-line hybrids, in zoomable PDF format. The top curves connect two parents of superior hybrids: blue, *IndII* restorers crossed with *IndI* male sterile lines; green, *IndII* restorers crossed with *IndII* male sterile lines; red, *IndI* restorers crossed with *IndI* male sterile lines; and yellow, *IndII* restorers crossed with male sterile lines. The nuclear genome phylogenetic tree in horizontal format is shown below the curves in the same color schema as in Fig. 1. Source data are provided as a Source Data file.

a

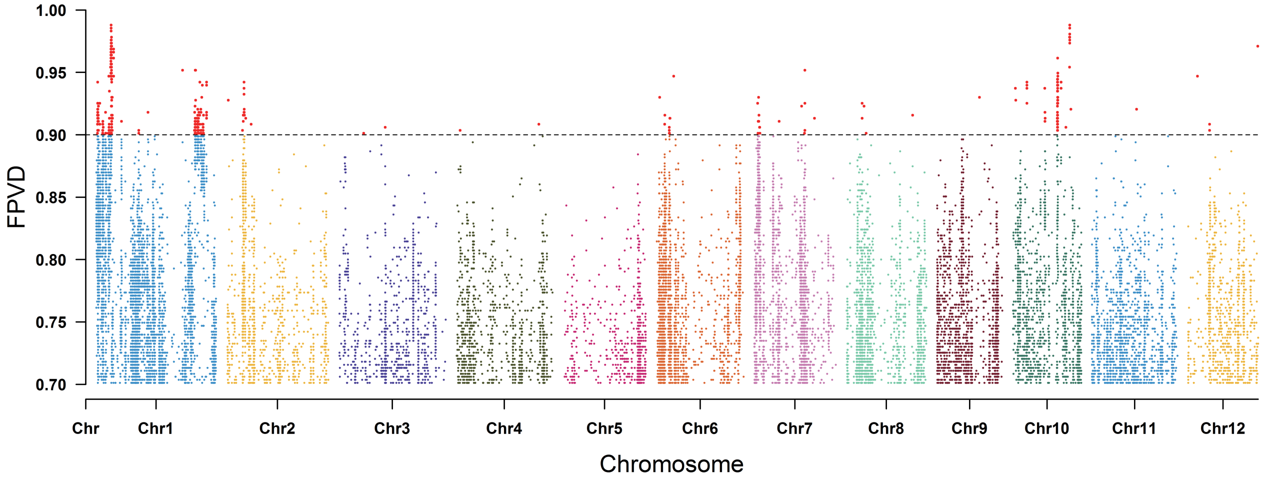

b

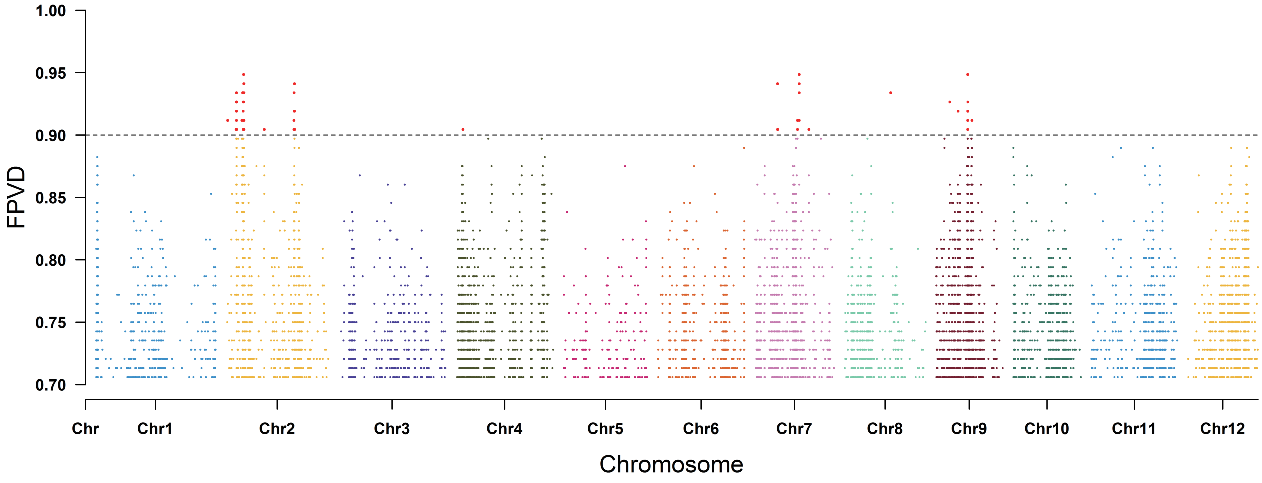

Supplementary Fig. 8 Genome-wide indel FPVD analyses. a, Superior 3-line hybrids (*n* = 415). **b**, Superior 2-line hybrids (*n* = 136). Source data are provided as a Source Data file.

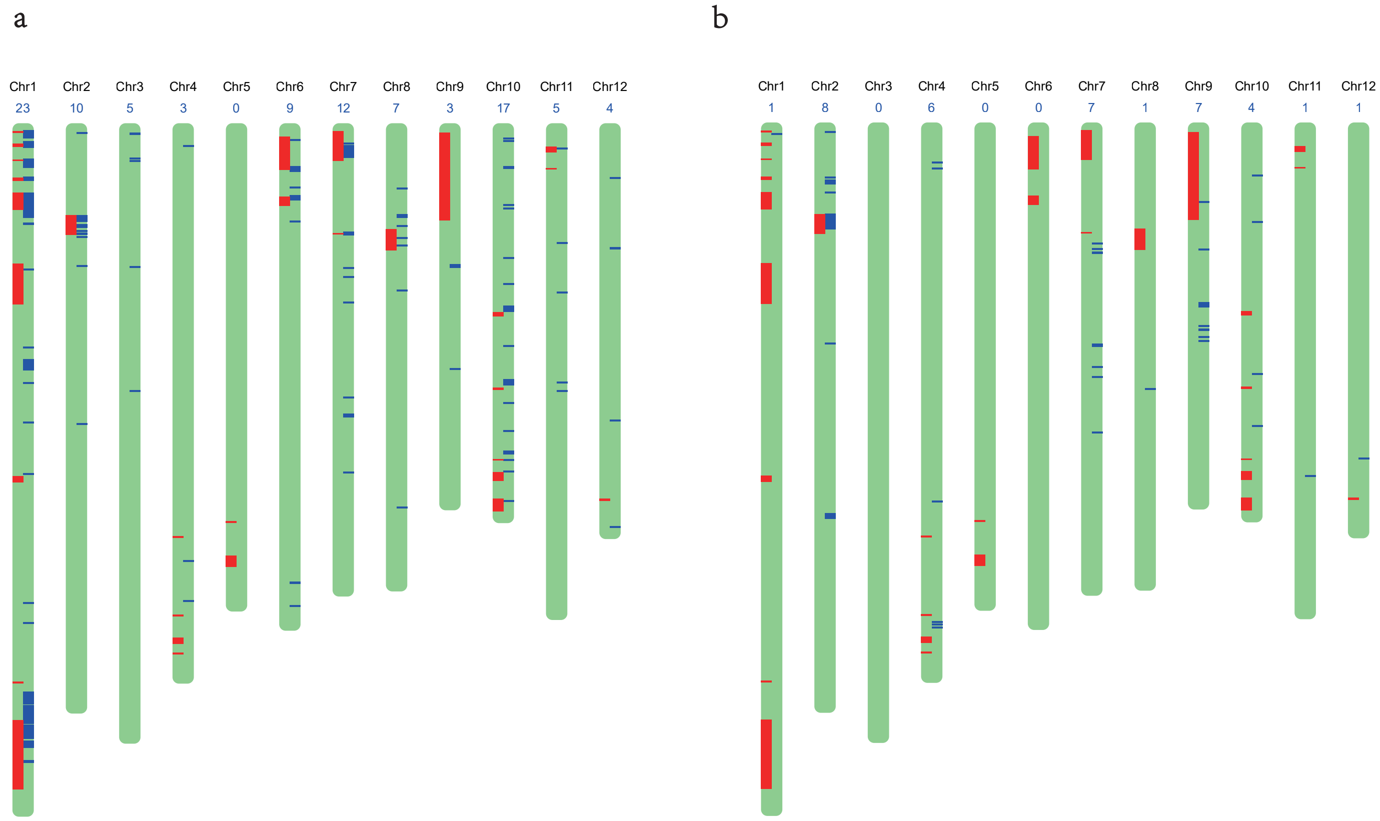

Supplementary Fig. 9 Comparison of 31 loci identified by Huang *et al.*[1] and the loci identified in this study. a, 98 loci identified in superior 3-line hybrids. **b**, 36 loci identified in superior 2-line hybrids. Red: 31 loci identified by Huang *et al*. Blue: loci identified in this study. The locations of the 31 loci identified by Huang *et al*. based on the Nipponbare genome were mapped to the R498 genome using BLAST, and some loci were extended due to short insertions in R498 genome. Source data are provided as a Source Data file.

a

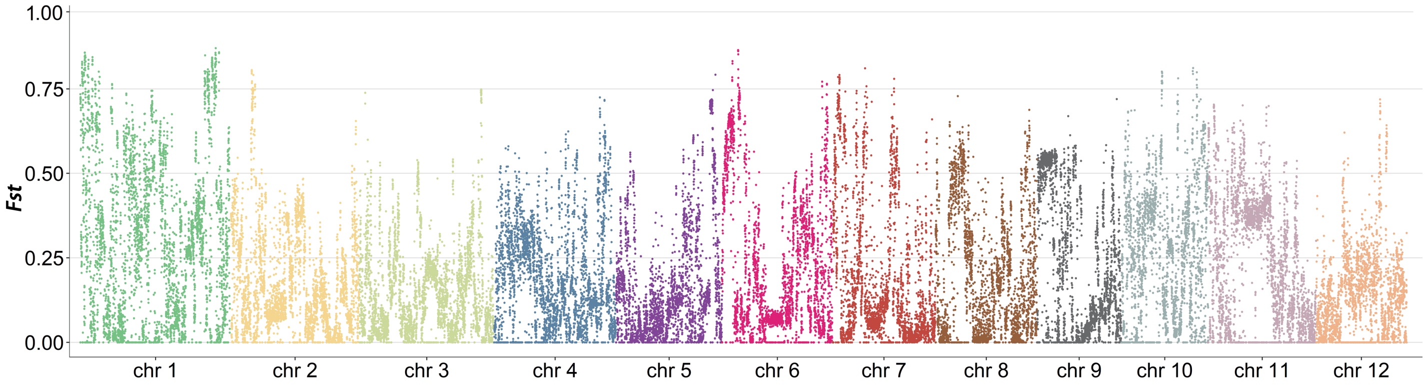

b

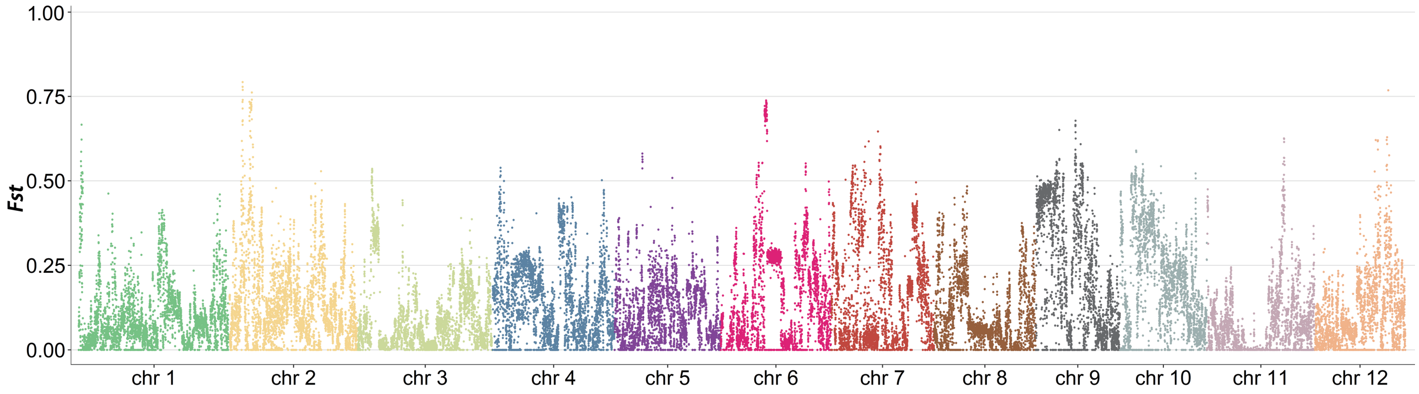

Supplementary Fig. 10 Fst plots for two parental populations. a, 3-line system: 211 CMS vs 294 3R accessions. **b**, 2-line system: 110 GMS vs 375 2R and 3R restorers. All genomic regions in 10-kb sliding windows were scanned in 10-kb steps, and the regions containing SNPs within the top 1% of the distribution that presented higher differentiation than expected were defined as having stronger signals of selection within the selective sweep regions. Source data are provided as a Source Data file.

Supplementary Table 1 Numbers and types of SNPs in the nuclear, mitochondrial, and chloroplast genomes of 1,143 *indica* accessions. R498 genomeswere used as the reference genomes. Syn: synonymous; nonsyn: nonsynonymous. Annotations were not performed for mitochondrial and chloroplast SNPs due to a lack of annotation information for the genomes of these two organelles.

chr	total	gene	mRNA	5'UTR	CDS	intron	3'UTR	inter-genic	syn	non-syn	stop codon gained	stop codon lost	start codon lost
Chr1	438,754	109,808	109,658	11,262	34,186	44,095	20,115	328,946	13,318	20,498	805	469	71
Chr2	380,060	92,440	92,296	9,705	26,997	37,930	17,664	287,620	10,492	16,359	621	305	45
Chr3	358,505	79,889	79,889	7,777	22,349	34,201	15,562	278,616	8,654	13,501	468	266	55
Chr4	320,946	73,404	73,404	7,465	23,513	30,200	12,226	247,542	8,820	14,277	616	341	46
Chr5	269,320	54,181	54,181	5,717	16,434	21,817	10,213	215,139	6,374	9,825	383	200	35
Chr6	317,406	69,060	69,060	6,828	22,219	27,425	12,588	248,346	8,682	13,346	494	248	30
Chr7	303,929	66,687	66,687	6,370	20,995	27,576	11,746	237,242	8,293	12,656	460	250	34
Chr8	325,494	67,549	67,549	6,666	20,662	28,424	11,797	257,945	8,064	12,242	482	229	47
Chr9	262,105	52,835	52,835	4,545	16,104	23,053	9,133	209,270	6,017	9,775	412	202	36
Chr10	256,078	51,847	51,847	4,594	16,919	21,952	8,382	204,231	6,672	10,106	362	171	21
Chr11	334,446	77,134	77,031	7,065	27,387	30,836	11,743	257,312	10,634	16,525	659	319	47
Chr12	293,506	61,397	61,397	6,028	20,094	24,711	10,564	232,109	7,888	11,969	435	260	31
nuclear total	3,860,549	856,231	855,834	84,022	267,859	352,220	151,733	3,004,318	103,908	161,079	6,197	3,260	498
mitochondria	452												
chloroplast	102												

Supplementary Table 2 Numbers and types of indels in the nuclear, mitochondrial, and chloroplast genomes of the 1,143 *indica* accessions. R498 genomes were used as reference genomes. Annotations were not performed for mitochondrial and chloroplast indels due to a lack of annotation information for these two organelle genomes

chr	total	gene	mRNA	5'UTR	CDS	intron	3'UTR	intergenic
Chr1	81,996	21,605	21,569	2,778	3,560	10,822	4,409	60,391
Chr2	70,414	17,414	17,394	2,250	3,019	8,600	3,525	53,000
Chr3	62,705	14,698	14,698	1,891	2,302	7,491	3,014	48,007
Chr4	62,848	15,088	15,088	1,881	2,963	7,561	2,683	47,760
Chr5	49,451	10,653	10,653	1,478	1,800	5,204	2,171	38,798
Chr6	55,228	12,711	12,711	1,686	2,356	6,018	2,651	42,517
Chr7	61,172	14,306	14,306	1,857	2,685	6,884	2,880	46,866
Chr8	59,284	13,131	13,131	1,791	2,375	6,591	2,374	46,153
Chr9	48,631	10,444	10,444	1,133	1,853	5,379	2,079	38,187
Chr10	45,023	9,642	9,642	1,118	1,805	4,932	1,787	35,381
Chr11	63,231	15,740	15,717	1,867	3,216	8,037	2,597	47,491
Chr12	56,962	12,691	12,691	1,615	2,298	6,479	2,299	44,271
nuclear total	716,994	168,123	168,044	21,345	30,232	83,998	32,469	548,871
mitochondria	30							
chloroplast	19							

Supplementary Table 3 Numbers of *IndI* and *IndII* individuals by subgroup for the 1,143 indica accessions.

group	*IndI*	*IndII*	total
cytoplasm male sterility (CMS)	186	25	211
3-line maintainer (3M)	13	2	15
3-line restorer (3R)	7	287	294
2-line genic male sterile (GMS)	21	89	110
2-line restorer (2R)	15	66	81
germplasm (GR)	8	128	136
conventional rice (CR)	143	153	296
total	393	750	1143

Supplementary Table 4 Number of hybrids resulting from specific crossing types in superior 3- and 2-line hybrid rice. (Male - Female).

hybrid type	total	*IndII-IndI*	*IndII-IndII*	*IndI-IndI*	*IndI-IndII*
3-line	415	382	28	5	0
2-line	136	30	95	9	2

Supplementary Table 5 Loci and genes identified based on an FPVD cut-off value of 0.9 in simulated "inferior" 3-line hybrids. The locus borders were determined as the 50 kb flanking regions on both sides of the SNPs/indels. The annotation data are from http://qtaro.abr.affrc.go.jp/, http://www.mbkbase.org/R498/, https://rapdb.dna.affrc.go.jp/, and http://rice.plantbiology.msu.edu.

FPVD locus				R498 locus			RAPDB locus	MSU locus	description
chr	start	end	length	locus	start	end			
Chr1	5490822	5590822	100000	OsR498G0100362800.01	5552148	5559540	Os01g0201900	LOC_Os01g10530	expressed protein
				OsR498G0100361800.01	5519296	5532066	Os01g0201700	LOC_Os01g10504	MADS3 - MADS-box family gene with MIKCc type-box, expressed
				OsR498G0100360800.01	5490048	5490941	Os01g0201500	LOC_Os01g10480	plastocyanin-like domain containing protein, putative, expressed
				OsR498G0100362200.01	5539632	5546374	Os01g0201800	LOC_Os01g10520	expressed protein
				OsR498G0100361000.01	5495939	5497381	Os01g0201600	LOC_Os01g10490	keratin, type I cytoskeletal 9, putative, expressed
Chr10	20987285	21121133	133848	OsR498G1018982300.01	21122086	21125678	Os10g0496900	LOC_Os10g35370	oxidoreductase, short chain dehydrogenase%2Freductase family domain containing family, expressed
				OsR498G1018976000.01	20997525	20997874	Os10g0495200	LOC_Os10g35240	Rf1, mitochondrial precursor, putative, expressed
				OsR498G1018980600.01	21107033	21107469	Os10g0496200	LOC_Os10g35310	expressed protein
				OsR498G1018982700.01	21128153	21136949	Os10g0497000	LOC_Os10g35380	expressed protein
				OsR498G1018979200.01	21075972	21081189	Os10g0495500	LOC_Os10g35280	nucleolar complex protein 2, putative, expressed
				OsR498G1018979800.01	21098909	21100377	Os10g0496000	LOC_Os10g35300	dof zinc finger domain containing protein, putative, expressed
				OsR498G1018975500.01	20991660	20995551	Os10g0495000	LOC_Os10g35220	KH domain containing protein, putative, expressed
				OsR498G1018972900.01	20956422	20958817	Os10g0494950	LOC_Os10g35210	expressed protein

FPVD locus				R498 locus			RAPDB locus	MSU locus	description
chr	start	end	length	locus	start	end			
				OsR498G1018980800.01	21107117	21108061	Os10g0496200	LOC_Os10g35310	expressed protein
				OsR498G1018973300.01	20960271	20967006	Os10g0495000	LOC_Os10g35220	KH domain containing protein, putative, expressed
				OsR498G1018979400.01	21081714	21093558	Os10g0495600	LOC_Os10g35290	DNA-directed RNA polymerase I subunit RPA2, putative, expressed
				OsR498G1018978600.01	21061380	21067065	Os10g0495300	LOC_Os10g35250	vacuolar protein-sorting protein bro1, putative, expressed
				OsR498G1018981000.01	21111132	21112271	Os10g0496400	LOC_Os10g35330	expressed protein
				OsR498G1018975000.01	20978809	20982664	Os10g0495200	LOC_Os10g35240	Rf4, Rf-4, fertility restorer 4
				OsR498G1018972800.01	20947344	20954703	Os10g0494800	LOC_Os10g35200	katanin p80 WD40 repeat-containing subunit B1 homolog 1, putative, expressed
				OsR498G1018982900.01	21138666	21142235	Os10g0497100	LOC_Os10g35390	acyltransferase, putative, expressed
				OsR498G1018983700.01	21158354	21163060	Os10g0497300	LOC_Os10g35436	Rf1, mitochondrial precursor, putative, expressed
				OsR498G1018972500.01	20943633	20947184	Os10g0494500	LOC_Os10g35190	ZOS10-06 - C2H2 zinc finger protein, expressed
				OsR498G1018978200.01	21056282	21059846	Os10g0495200	LOC_Os10g35240	Rf1, mitochondrial precursor, putative, expressed
				OsR498G1018983100.01	21147321	21155261	Os10g0497300	LOC_Os10g35436	Rf1, mitochondrial precursor, putative, expressed
				OsR498G1018979500.01	21095215	21098453	Os10g0495900	LOC_Os10g35294	fiber protein Fb34, putative, expressed
				OsR498G1018981900.01	21119816	21120959	None	LOC_Os10g35360	expressed protein
				OsR498G1018978800.01	21069125	21073025	Os10g0495200	LOC_Os10g35240	Rf1, mitochondrial precursor, putative, expressed

FPVD locus				R498 locus			RAPDB locus	MSU locus	description
chr	start	end	length	locus	start	end			
				OsR498G1018973800.01	20967681	20968908	Os10g0495200	LOC_Os10g35240	Rf1, mitochondrial precursor, putative, expressed
				OsR498G1018972100.01	20934009	20940142	Os10g0494300	LOC_Os10g35180	white-brown complex homolog protein 11, putative, expressed

Supplementary Reference

1. Huang X, *et al*. Genomic analysis of hybrid rice varieties reveals numerous superior alleles that contribute to heterosis. *Nat Commun* **6**, 6258 (2015).
