# Supplementary material for: Resequencing of 1,143 indica rice accessions reveals important genetic variations and different heterosis patterns
Source: Nat Commun. 2020 Sep 22;11:4778. doi: 10.1038/s41467-020-18608-0 (PMC7508829; doi:10.1038/s41467-020-18608-0)
Supplement: Supplementary file 3 — Reporting Summary [file 41467_2020_18608_MOESM3_ESM.pdf]

nature research

Corresponding author(s): Dingyang Yuan

Last updated by author(s): Aug 18, 2020

Reporting Summary

Nature Research wishes to improve the reproducibility of the work that we publish. This form provides structure for consistency and transparency in reporting. For further information on Nature Research policies, see Authors & Referees and the Editorial Policy Checklist .

Statistics

For all statistical analyses, confirm that the following items are present in the figure legend, table legend, main text, or Methods section.

n/a	Confirmed	
☐	☒	The exact sample size (*n*) for each experimental group/condition, given as a discrete number and unit of measurement
☐	☒	A statement on whether measurements were taken from distinct samples or whether the same sample was measured repeatedly
☐	☒	The statistical test(s) used AND whether they are one- or two-sided<br>*Only common tests should be described solely by name; describe more complex techniques in the Methods section.*
☒	☐	A description of all covariates tested
☒	☐	A description of any assumptions or corrections, such as tests of normality and adjustment for multiple comparisons
☐	☒	A full description of the statistical parameters including central tendency (e.g. means) or other basic estimates (e.g. regression coefficient) AND variation (e.g. standard deviation) or associated estimates of uncertainty (e.g. confidence intervals)
☐	☒	For null hypothesis testing, the test statistic (e.g. *F*, *t*, *r*) with confidence intervals, effect sizes, degrees of freedom and *P* value noted<br>*Give P values as exact values whenever suitable.*
☒	☐	For Bayesian analysis, information on the choice of priors and Markov chain Monte Carlo settings
☒	☐	For hierarchical and complex designs, identification of the appropriate level for tests and full reporting of outcomes
☒	☐	Estimates of effect sizes (e.g. Cohen's *d*, Pearson's *r*), indicating how they were calculated

Our web collection on statistics for biologists contains articles on many of the points above.

Software and code

Policy information about availability of computer code

Data collection	No software was used
Data analysis	BWA v0.7.8, GATK v3.8, BEDtools v2.26.0, R version v3.2.3, APE7 v4.1, iTOL, PLINK v1.90, GEMMA v0.98.1-0, Cytoscape v3.6.0, ADMIXTURE v1.3.0, BLAST v2.6.0, SAMTools v1.7, JBrowse v1.12.3

For manuscripts utilizing custom algorithms or software that are central to the research but not yet described in published literature, software must be made available to editors/reviewers. We strongly encourage code deposition in a community repository (e.g. GitHub). See the Nature Research guidelines for submitting code & software for further information.

Data

Policy information about availability of data

All manuscripts must include a data availability statement. This statement should provide the following information, where applicable:

- Accession codes, unique identifiers, or web links for publicly available datasets
- A list of figures that have associated raw data
- A description of any restrictions on data availability

All resequencing read data have been deposited at NCBI with BioProject ID PRJNA656900. The source data for Figs. 1a, b, 2b-e, 3a-d, 4c, d, and Supplementary Figs. 1-10 are provided as a Source Data file. All other data are available from the authors upon reasonable request.

Field-specific reporting

Please select the one below that is the best fit for your research. If you are not sure, read the appropriate sections before making your selection.

☒ Life sciences ☐ Behavioural & social sciences ☐ Ecological, evolutionary & environmental sciences

Life sciences study design

All studies must disclose on these points even when the disclosure is negative.

Sample size	Indica rice accession sample size was chosen based on indica resources demonstrating comprehensive, diverse, and important resources in China. Sample size 3 was chosen for PCR and Cd concentration measurement to ensure reproducibility. All other sample sizes were sample totals so as to maximize the sample sizes and avoid random sampling.
Data exclusions	No data were excluded from the analyses
Replication	Three biological replicates were analyzed for PCR and Cd concentration measurement to ensure reproducibility across experiments
Randomization	Randomization and covariates were not relevant to our study design as we investigated single factors within each study.
Blinding	Blinding was not relevant as all processing methods were done through available software with consistent parameters utilized across all groups.

Reporting for specific materials, systems and methods

We require information from authors about some types of materials, experimental systems and methods used in many studies. Here, indicate whether each material, system or method listed is relevant to your study. If you are not sure if a list item applies to your research, read the appropriate section before selecting a response.

Materials & experimental systems

n/a	Involved in the study
☒	☐ Antibodies
☒	☐ Eukaryotic cell lines
☒	☐ Palaeontology
☒	☐ Animals and other organisms
☒	☐ Human research participants
☒	☐ Clinical data

Methods

n/a	Involved in the study
☒	☐ ChIP-seq
☒	☐ Flow cytometry
☒	☐ MRI-based neuroimaging
