# Supplementary material for: Resequencing of 1,143 indica rice accessions reveals important genetic variations and different heterosis patterns
Source: Nat Commun. 2020 Sep 22;11:4778. doi: 10.1038/s41467-020-18608-0 (PMC7508829; doi:10.1038/s41467-020-18608-0)
Supplement: Supplementary file 4 — Description of Additional Supplementary Files [file 41467_2020_18608_MOESM4_ESM.pdf]

Description of Additional Supplementary Files

File Name: Supplementary Data 1
Description: List of 1,143 *indica* rice accessions used in this study

File Name: Supplementary Data 2
Description: Sequencing depths and mapping rates of the 1,143 *indica* rice accessions. The R498 nuclear genome size (437,699,851 bp) was used for sequencing coverage depth calculation

File Name: Supplementary Data 3
Description: Kinship relationships between accessions with kinship coefficient greater than 0.45

File Name: Supplementary Data 4
Description: List of superior indica crosses whose parents were resequenced in this study. Source: www.ricedata.cn

File Name: Supplementary Data 5
Description: Loci and genes associated with heterosis identified based on an FPVD cut-off value of 0.9 in superior 3-line hybrids. The locus borders were determined as the 50 kb flanking regions on both sides of the SNPs/indels. The annotation data are from http://qtaro.abr.affrc.go.jp/, http://www.mbkbase.org/R498/, https://rapdb.dna.affrc.go.jp/, and http://rice.plantbiology.msu.edu/.

File Name: Supplementary Data 6
Description: Loci and genes associated with heterosis identified based on an FPVD cut-off value of 0.9 in superior 2-line hybrids. The locus borders were determined as the 50 kb flanking regions on both sides of the SNPs/indels. The annotation data are from http://qtaro.abr.affrc.go.jp/, http://www.mbkbase.org/R498/, https://rapdb.dna.affrc.go.jp/, and http://rice.plantbiology.msu.edu/.

File Name: Supplementary Data 7
Description: Loci and genes identified based on an FPVD cut-off value of 0.9 in simulated "inferior" 2-line hybrids. The locus borders were determined as the 50 kb flanking regions on both sides of the SNPs/indels. The annotation data are from http://qtaro.abr.affrc.go.jp/, http://www.mbkbase.org/R498/, https://rapdb.dna.affrc.go.jp/, and http://rice.plantbiology.msu.edu/.

File Name: Supplementary Data 8
Description: List of loci and genes covered with top 1% F_{st} value windows for the 3-line system. Some R498 loci are redundant if they are located on multiple Fst windows. The annotation data are from http://qtaro.abr.affrc.go.jp/,

http://www.mbkbase.org/R498/, https://rapdb.dna.affrc.go.jp/, and http://rice.plantbiology.msu.edu/.

File Name: Supplementary Data 9
Description: List of loci and genes covered with top 1% F_{st} value windows for the 2-line system. Some R498 loci are redundant if they are located on multiple Fst windows. The annotation data are from http://qtaro.abr.affrc.go.jp/, http://www.mbkbase.org/R498/, https://rapdb.dna.affrc.go.jp/, and http://rice.plantbiology.msu.edu/.

File Name: Supplementary Data 10
Description: List of loci and genes that we studied and top 10 accessions with the lowest gene coverages in brackets. The loci with the gene coverage below 10% are highlighted.
