# Supplementary material for: Resequencing of 1,143 indica rice accessions reveals important genetic variations and different heterosis patterns
Source: Nat Commun. 2020 Sep 22;11:4778. doi: 10.1038/s41467-020-18608-0 (PMC7508829; doi:10.1038/s41467-020-18608-0)
Supplement: Supplementary file 2 — Peer Review [file 41467_2020_18608_MOESM2_ESM.pdf]

Reviewers' comments:

Reviewer #1 (Remarks to the Author):

The manuscript described resequencing and analysis of 1,143 indica rice accessions, most of which were parental lines in hybrid rice breeding. The work provided an important resource for genetic studies and breeding in hybrid rice. The genome data may be useful in genetic improvements of parental lines for the three-line and two-line indica hybrid rice. However, many findings in this paper have been reported in the previous papers. The data could be re-analyzed to find something new, for example, the digital pedigree relationship of the parental lines covering the last fifty years. Moreover, the figures were not well prepared and the manuscript was not clearly written (with many grammar errors).
Below are some minor suggestions for the manuscript.

1. The significances and novelties of the work should be stated in the Abstract section.
2. Considering that "the elite accessions were widely shared and further modified for improvement in different ecotypes" in line 74, this collection could be analyzed to tell a story on the hybrid breeding selections over the last fifty years, which would be useful and interesting to the rice community.
3. More details on the 1,143 indica rice accessions (e.g., hybrid rice that the accessions generated) should be listed in the Supp Tables.
4. In this work, a new approach was used to identify the heterotic loci by FPVD which largely overlaps 31 highly differentiated loci discovered by Huang et al. Any new loci identified here?
5. OsNramp5 was identified through PAV, and a knock-out material was used for functional validation. More details should be added, e.g.: CRISPR or T-DNA, the mutation sites, the change of expression level.
6. In Line 192, it was stated "there is a 408 kb deletion on chromosome 7". Any experimental data to support it? It may result from misalignment or short-read sequencing problems.
7. Most figures were not clear and need to be re-prepared (e.g., Fig4 may be prepared from screenshots from some Genome Browse software).

Reviewer #2 (Remarks to the Author):

Lv et al have sequenced the largest collection of indica rice accessions mostly related to hybrid rice to date. The indica reference quality genome of R498 was used to call variants, enabling a more complete analysis than if the Nipponbare temperate japonica genome had been used. They present a novel method for identifying regions important for heterosis based on the frequency of parental variant differences and identify regions that carry important genes in the 2-line and 3-line systems. PAV analysis showed that an important locus for Cd uptake was missing from two accessions and that knockouts had reduced Cd uptake. This result has implication for breeding for this important trait.

There are a few points that need clarification and editing:

1) On line 73, three indica ecotypes are referred to, but the ecotypes are defined in terms of regions in the Fig. legend. This should be indicated in the text.

2) Also, on lines 76 and 77, two subpopulations are defined without referring to the method is only stated in the supplement where Admixture at K2 was used to partition into these two subpopulations. While the dendrogram presented in Fig 1a does show a split into two subpopulations it could also be viewed as showing 3, 4 or 5 subpopulations. Were Admixture tests at higher K values tested? Please explain and justify apart from splitting into groups based on relatedness to Zhenshan97 and Minghui63.

Minor points:

Line 63: MBKbase not mbkbase.

Line 204. Was the OGRO database associated with QTARO also used? If so, cite OGRO here.

References need to be checked: "project, r.g." looks strange as the author.

FIg 2. Visibility would be improved if the height of the tree and thickness of the subpopulation bar below are increased.

Supplementary Table 1: "Accessions" are actually variety names. The table could be improved by indicating the sources and any genebank accession numbers.

Reviewer #3 (Remarks to the Author):

The authors report a major effort to describe the heterosis patterns in Xian (indica) hybrids of China by analyzing genome sequences of 1,143 parental lines of common Xian hybrids in China. The huge amounts of genomic data, when analyzed and interpreted properly, are expected to provide valuable information on the population organization of the hybrid parents and for future improvement of hybrid rice. Unfortunately, there were some technical flaws in their data analyses and result summary which would have seriously affected their conclusions. Thus, the current form of the MS does not meet the high standard for publication in NC and major revision and additional analyses are required. These technical problems are given as follows:

1. The separation of all hybrid parents into the 3-line and 2-line groups as heterosis groups was incorrect because these two groups of hybrids differ for their male sterility genetic systems, but not necessarily for the levels of heterosis. Loci contributing to heterosis of a two-line hybrid can also contribute to heterosis of a 3-line hybrid. Their results that those loci differentiating the two-line parents and those differentiating the 3-line parents included their respective CMS gene and the GMS gene, would imply that many of those so-called heterosis genes are actually related to their MS systems (see the comment 3). Thus, their conclusion that 3-line and 2-line hybrids involved different loci and genes for heterosis is not valid, because this conclusion would be true for any two unrelated hybrids.

2. The authors should not use the two previously defined groups of Xian accessions, IndicaI and IndicaII, in their phylogenetic analyses and result description unless a significant portion of their materials are shared with those used by Huang et al., or their result clearly classified the parental lines into the same two groups. This problem was clearly reflected by the choice of K=2 in their phylogenetic analysis. In fact, they should compare the results for K=2, 3, 4…, and find at which value of k would best describe the population structure of the hybrid parents in China and then classify the parental lines and their hybrids accordingly. This does not mean they should not compare the two-line hybrids and 3-line hybrids. Instead, by doing so would help them to better interpret their results.

3. The use of FPVD by the authors as the key parameter to identify genes/loci potentially contributing to heterosis was reasonable. However, their selection of 90% FPVD as the cutoff line for identification of loci associated with heterosis was arbitrary and problematic. In fact, the authors should use the percentages of FPVD (the average levels of genomewide heterozygosity) of individual hybrids (crosses that were commercial hybrids) to classify individual hybrids and see if there are specific patterns regarding the contributions of individual hybrid parents. More informative results of this analysis can

be obtained if the gene CDS haplotypes and gene PAVs, in addition to random SNPs and InDels, are used as the input data for calculating FPVD and the combined results should be summarized together in the same fig. across 12 chromosomes.

4. Given the huge number of potential hybrids that can be generated from crosses between the parents (between all 3-line restorers x all CMS lines, between all restorers x all GMS lines), an important question would be why only 551 hybrids were commercially released in China from these large numbers of parents. Then, pairwise comparisons between the commercial hybrids and those unreleased hybrids in each group (such as 3-line restorers x 3-line CMS) can be made for different parameters such as FPVD, Fst, and specific set of genes/loci, etc. might provide useful information regarding why these commercial hybrids were more superior.

5. Similarly, the authors' choices of relatively few genes/loci for in-depth analyses were arbitrary. Instead, they should use those of the 3200+ cloned rice genes which are related to yield traits and have large-effect SNPs and InDels among the sequenced hybrid parents.

6. In China, Xian and Geng, instead of indica and japonica, has been used for naming the two subspecies of rice for more than 1,500 years until today and the naming system of indica and japonica is misleading and incorrect, as clearly shown by the recent paper of 3,010 rice genomes in Nature. The reviewer suggests the authors should use Xian, instead of Indica, in their MS.

7. The reviewer is wondering why the authors performed GWAS for 7 traits without grain yield itself and how the GWAS results contribute to their key conclusion(s). There was no detailed description on how the phenotyping experiments were conducted in Changsha and Sanya with regard to the number of replications, experiment design, trait measurements, etc.

8. Finally, the way by which the MS was written was awkward. The authors put the introduction, results, conclusion and discussion together in the text of their MS such that a very large portion of the MS was to describe previously published results related to their results, leaving very limited spaces to describe, interpret and summarize their own results, given the large amounts of data, results and information from their analyses. This makes it difficult for readers to find what are the key and novel discoveries from their research.

Reviewer #4 (Remarks to the Author):

This study by Lv et al. aimed as they say in the abstract of their study L39 "... goals were to obtain complete genetic variation information for these accessions and to comprehensively identify the key gene loci for heterosis of hybrid rice...". They genome sequenced >1000 Indica parental lines that were used in hybrid varieties to answer this goal. However reading the manuscript I'm not sure which analysis leads to the insight on the loci responsible for heterosis.
In my mind I thought heterosis is simply the effect of heterozygous genotypes as they were formed from parents with different genotypes. The study they cite in the manuscript (Huang et al 2015 Nat comm) had basically done what the authors set out to answer as they indicate in the abstract. Here in this manuscript they simply look at allele frequency differences of the parents which the frequency difference of itself has really nothing to do with heterosis per se. There's no analysis that sets out to see if those parental alleles are responsible for heterosis.
What they chose to do is simply look at sites where the parents have very different allele frequencies. First I wasn't sure how the parents were grouped to calculate the allele frequency. In fact the M&M is so sparse that its hard to see the details of their method. For instance how do they deal with the fact that not all polymorphic sites will have the same allele count because of missing genotype calls naturally arising from next gene sequencing data? How much of the difference is due to simply the underlying population structure and hence has no heterosis effect when brought together. No direct

connection between heterosis sites and parental genotypes makes this study not what it set out to do in its title and abstract.
Also there are some contents of the manuscript where as a reader wonders why its there. For instance the whole half of this manuscript deals with explaining the genetic relationships of the parents. This is a pretty short study but half just simply explaining who's related to who seems a waste of a space especially when its not really that relevent to their main topic.
The Cd tolerance, PAV analysis, and GWAS results are also not so relevent to the manuscript as they really have nothing to do with heterosis either. Why were they there for?
Lastly the result they present are essentially for the most validating previous studies. The whole study reads like a short validation study. The dataset they generate is great but the analysis doesn't seem to be on par with what they set out to do. In fact this type of manuscripts frankly I've read more frequently in a more specialized journal like Rice.

The reviewer's remarks are in black. Author's reply is in blue.

Reviewer #1 (Remarks to the Author):

General reply:

We revised the manuscript, upon suggestions from the editor and reviewers. The major changes are:

1. It is in the Article format now. Previously it was in the Nature Genetics Letter format.
2. We did try to do digital pedigree studies as you suggested, but it turned out to be too difficult for the following reasons: 1: the pedigree information of many accessions was not well documented or is not available; 2: many accessions that were used to develop the accessions in this study have not been resequenced; 3: many accessions in this study are very close to each other in relationships, making it hard to determine the parent-child relationships. So instead, we performed the kinship analysis and think that our results revealed some important kinship information among these 1,143 indica accessions, such as 98 3R, 12 2R, and 3 CR accessions among 1,143 accessions in this study have relationship with a 0.45+ kinship coefficient.
3. We have performed the FPVD analyses on the simulated "inferior" 3- and 2-line hybrids (to mimic the unreleased hybrids), and have added the results to the manuscript. They can serve as controls for analyses of superior (released) hybrids.
4. Most figures are in zoomable high definition PDF format now, except the screenshots in the manuscript file in MS-Word

The replies to your specific questions and suggestions are as follow (See the text in blue)

The manuscript described resequencing and analysis of 1,143 indica rice accessions, most of which were parental lines in hybrid rice breeding. The work provided an important resource for genetic studies and breeding in hybrid rice. The genome data may be useful in genetic improvements of parental lines for the three-line and two-line indica hybrid rice. However, many findings in this paper have been reported in the previous papers. The data could be re-analyzed to find something new, for example, the digital pedigree relationship of the parental lines covering the last fifty years. Moreover, the figures were not well prepared and the manuscript was not clearly written (with many grammar errors).
Below are some minor suggestions for the manuscript.

1. The significances and novelties of the work should be stated in the Abstract section.

Thanks for your suggestion! The original format was in the letter format. We have now changed to the article format with a formal abstract.

2. Considering that "the elite accessions were widely shared and further modified for improvement in different ecotypes" in line 74, this collection could be analyzed to tell a story on the hybrid breeding selections over the last fifty years, which would be useful and interesting to the rice community.

Thank for your suggestion. We have performed kinship analysis in the 1,143 accessions, and hope that it helps. Please also see Point 2 in the general reply.

3. More details on the 1,143 indica rice accessions (e.g., hybrid rice that the accessions generated) should be listed in the Supp Tables.
Thanks for your suggestion! The information is now in Supplementary Table 6.

4. In this work, a new approach was used to identify the heterotic loci by FPVD which largely overlaps 31 highly differentiated loci discovered by Huang et al. Any new loci identified here?
Among 98 loci we identified for the 3-line superior hybrids, 69 loci are new and do not overlap with loci identified by Dr. Huang.

5. OsNramp5 was identified through PAV, and a knock-out material was used for functional validation. More details should be added, e.g.: CRISPR or T-DNA, the mutation sites, the change of expression level.
Thanks for your suggestion. We have added more details in Lines 327-329

6. In Line 192, it was stated “there is a 408 kb deletion on chromosome 7”. Any experimental data to support it? It may result from misalignment or short-read sequencing problems.
The conclusion was based on the read/reference genome alignment. We checked all other accessions and none of them had this big deletion in this region. Only Luohong3A and Luohong4A have this big deletions and deletion patterns of in this region of these two accessions are very similar. Our resequencing coverage depth was about 17x, higher than many of the previous resequencing effort. So it is less likely that it was due to the misalignment or low NGS coverage problems.

7. Most figures were not clear and need to be re-prepared (e.g., Fig4 may be prepared from screenshots from some Genome Browse software).
Thanks for your suggestion! We have re-made all the figures and they are now in zoomable vectored or high definition PDF format.

The reviewer's remarks are in black. Author's reply is in blue.

Reviewer #2 (Remarks to the Author):

General reply:

We revised the manuscript, upon suggestions from the editor and reviewers. The major changes are:

1. It is in the Article format now. Previously it was in the Nature Genetics Letter format.
2. We did try to do digital pedigree studies as reviewer 1 suggested, but it turned out to be too difficult for the following reasons: 1: the pedigree information of many accessions was not well documented or is not available; 2: many accessions that were used to develop the accessions in this study have not been resequenced; 3: many accessions in this study are very close to each other in relationships, making it hard to determine the parent-child relationships. So instead, we performed the kinship analysis and think that our results revealed some important kinship information among these 1,143 indica accessions, such as 98 3R, 12 2R, and 3 CR accessions among 1,143 accessions in this study have relationship with a 0.45+ kinship coefficient.
3. We have performed the FPVD analyses on the simulated "inferior" 3- and 2-line hybrids (to mimic the unreleased hybrids), and have added the results to the manuscript. They can serve as controls for analyses of superior (released) hybrids.
4. Most figures are in zoomable high definition PDF format now, except the screenshots in the manuscript file in MS-Word

The replies to your specific questions and suggestions are as follow (See the text in blue)

Lv et al have sequenced the largest collection of indica rice accessions mostly related to hybrid rice to date. The indica reference quality genome of R498 was used to call variants, enabling a more complete analysis than if the Nipponbare temperate japonica genome had been used. They present a novel method for identifying regions important for heterosis based on the frequency of parental variant differences and identify regions that carry important genes in the 2-line and 3-line systems. PAV analysis showed that an important locus for Cd uptake was missing from two accessions and that knockouts had reduced Cd uptake. This result has implication for breeding for this important trait.

Thanks for reading our manuscript and providing the valuable feedbacks!

There are a few points that need clarification and editing:

1) On line 73, three indica ecotypes are referred to, but the ecotypes are defined in terms of regions in the Fig. legend. This should be indicated in the text.

Thanks for your suggestion! We have modified as you suggested by moving it to the introduction to Lines 81-82, and changed "ecotypes" to "environments".

2) Also, on lines 76 and 77, two subpopulations are defined without referring to the method is only stated in the supplement where Admixture at K2 was used to partition into these two subpopulations. While the dendrogram presented in Fig 1a does show a split into two

subpopulations it could also be viewed as showing 3, 4 or 5 subpopulations. Were Admixture tests at higher K values tested? Please explain and justify apart from splitting into groups based on relatedness to Zhenshan97 and Minghui63.
Thanks for your suggestion! We are now showing the population structures for *K* values from 2 to 6.

Minor points:

Line 63: MBKbase not mbkbase.
We have changed as the reviewer suggested.

Line 204. Was the OGRO database associated with QTARO also used? If so, cite OGRO here.
Thanks for your suggestions! Since other reviewers said that there were no new findings in the GWAS results, we have deleted the GWAS part.

References need to be checked: "project, r.g." looks strange as the author.
Thanks for your suggestions! This was due to an issue in the EndNote software. We have manually corrected.

FIg 2. Visibility would be improved if the height of the tree and thickness of the subpopulation bar below are increased.
Thanks for your suggestions! We have tried to increase the height of the tree but it seemed not helpful. However, we have made the diagram a zoomable PDF, and also providing a separate PDF file as supplementary figures.

Supplementary Table 1: "Accessions" are actually variety names. The table could be improved by indicating the sources and any genebank accession numbers.
Thanks for pointing it out! We have changed from “Accession” to “variety name”.

The reviewer’s remarks are in black. Author’s reply is in blue.

Reviewer #3 (Remarks to the Author):

General reply:

We revised the manuscript, upon suggestions from the editor and reviewers. The major changes are:

1. It is in the Article format now. Previously it was in the Nature Genetics Letter format.
2. We did try to do digital pedigree studies as reviewer 1 suggested, but it turned out to be too difficult for the following reasons: 1: the pedigree information of many accessions was not well documented or is not available; 2: many accessions that were used to develop the accessions in this study have not been resequenced; 3: many accessions in this study are very close to each other in relationships, making it hard to determine the parent-child relationships. So instead, we performed the kinship analysis and think that our results revealed some important kinship information among these 1,143 indica accessions, such as 98 3R, 12 2R, and 3 CR accessions among 1,143 accessions in this study have relationship with a 0.45+ kinship coefficient.
3. We have performed the FPVD analyses on the simulated “inferior” 3- and 2-line hybrids (to mimic the unreleased hybrids) as you suggested, and have added the results to the manuscript. They can serve as controls for analyses of superior (released) hybrids.
4. Most figures are in zoomable high definition PDF format now, except the screenshots in the manuscript file in MS-Word

The replies to your specific questions and suggestions are as follow (See the text in blue)

The authors report a major effort to describe the heterosis patterns in Xian (indica) hybrids of China by analyzing genome sequences of 1,143 parental lines of common Xian hybrids in China. The huge amounts of genomic data, when analyzed and interpreted properly, are expected to provide valuable information on the population organization of the hybrid parents and for future improvement of hybrid rice. Unfortunately, there were some technical flaws in their data analyses and result summary which would have seriously affected their conclusions. Thus, the current form of the MS does not meet the high standard for publication in NC and major revision and additional analyses are required. These technical problems are given as follows:

1. The separation of all hybrid parents into the 3-line and 2-line groups as heterosis groups was incorrect because these two groups of hybrids differ for their male sterility genetic systems, but not necessarily for the levels of heterosis. Loci contributing to heterosis of a two-line hybrid can also contribute to heterosis of a 3-line hybrid. Their results that those loci differentiating the two-line parents and those differentiating the 3-line parents included their respective CMS gene and the GMS gene, would imply that many of those so-called heterosis genes are actually related to their MS systems (see the comment 3). Thus, their conclusion that 3-line and 2-line hybrids involved different loci and genes for heterosis is not valid, because this conclusion would be true for any two unrelated hybrids.

Thanks for reading our manuscript and your suggestions! We agree with you that 3- and 2-line hybrids could share the same heterosis loci, theoretically. However, the reality is that they probably have different heterosis loci as both our study and a previous study (Huang *et al*, 2016, Nature 537:629-633) suggested, although ours and Huang's differ in loci number and locations----we believe that ours is more reliable because our results were based on a total of 551 hybrids, much more than 15 *indica-indica* hybrids used in Huang's study. Probably the different 3- and 2-line breeding requirements and histories caused such a difference. In the future, it would be nice if the different heterosis loci can be integrated together.

2. The authors should not use the two previously defined groups of Xian accessions, IndicaI and IndicaII, in their phylogenetic analyses and result description unless a significant portion of their materials are shared with those used by Huang et al., or their result clearly classified the parental lines into the same two groups. This problem was clearly reflected by the choice of K=2 in their phylogenetic analysis. In fact, they should compare the results for K=2, 3, 4…, and find at which value of k would best describe the population structure of the hybrid parents in China and then classify the parental lines and their hybrids accordingly. This does not mean they should not compare the two-line hybrids and 3-line hybrids. Instead, by doing so would help them to better interpret their results.
Thanks for your suggestion! We classified our indica accessions into *IndI* and *IndII* because they do fit into these two types well. We have also added population structure results with K values from 2-6.

3. The use of FPVD by the authors as the key parameter to identify genes/loci potentially contributing to heterosis was reasonable. However, their selection of 90% FPVD as the cutoff line for identification of loci associated with heterosis was arbitrary and problematic. In fact, the authors should use the percentages of FPVD (the average levels of genomewide heterozygosity) of individual hybrids (crosses that were commercial hybrids) to classify individual hybrids and see if there are specific patterns regarding the contributions of individual hybrid parents. More informative results of this analysis can be obtained if the gene CDS haplotypes and gene PAVs, in addition to random SNPs and InDels, are used as the input data for calculating FPVD and the combined results should be summarized together in the same fig. across 12 chromosomes.
Thanks for your suggestion! In the revised manuscript, we added the FPVD analysis results on the simulated "inferior" hybrids. We believe that the FPVD method did work in identifying the heterosis loci: it correctly picked up the Rf4 in 3-line, and tms5 in 2-line, which are involved in the fertility in 3 and 2-line systems, in both superior hybrids and simulated "inferior" hybrids.

4. Given the huge number of potential hybrids that can be generated from crosses between the parents (between all 3-line restorers x all CMS lines, between all restorers x all GMS lines), an important question would be why only 551 hybrids were commercially released in China from these large numbers of parents. Then, pairwise comparisons between the

commercial hybrids and those unreleased hybrids in each group (such as 3-line restorers x 3-line CMS) can be made for different parameters such as FPVD, Fst, and specific set of genes/loci, etc. might provide useful information regarding why these commercial hybrids were more superior.
Thanks for your suggestion! Although there is no public records or official documentation, we do know that many hybrids, if not all possible hybrids, between 3R and CMS for 3-line, 3R+2R and GMS for 2-line, have been tested in China. Most were not released because their performance did not satisfy the breeders. We have tried the FPVD analysis on the simulated “interior” hybrids, i.e., all possible hybrids with the real superior hybrids excluded, the results did show only the genetic differences related to the sterility is above the cut-off line 0.9.

5. Similarly, the authors’ choices of relatively few genes/loci for in-depth analyses were arbitrary. Instead, they should use those of the 3200+ cloned rice genes which are related to yield traits and have large-effect SNPs and InDels among the sequenced hybrid parents.
Thanks for your suggestion. We chose those genes/loci because they are most shared differences between the superior hybrids, thus they are most likely to be related to the heterosis. The FPVD analysis on the simulated “interior” hybrids showed that frequency of the differences these genetic variations and loci are not as high as in superior hybrids, validating our rationale.

6. In China, Xian and Geng, instead of indica and japonica, has been used for naming the two subspecies of rice for more than 1,500 years until today and the naming system of indica and japonica is misleading and incorrect, as clearly shown by the recent paper of 3,010 rice genomes in Nature. The reviewer suggests the authors should use Xian, instead of Indica, in their MS.
Thanks for your suggestion! We chose using “*indica*” because rice research community has been using this naming system. It is just easier to communicate with each other this way.

7. The reviewer is wondering why the authors performed GWAS for 7 traits without grain yield itself and how the GWAS results contribute to their key conclusion(s). There was no detailed description on how the phenotyping experiments were conducted in Changsha and Sanya with regard to the number of replications, experiment design, trait measurements, etc.
Thanks for your suggestion! Because there is no new finding in our GWAS results, we have removed GWAS related parts from our manuscript.

8. Finally, the way by which the MS was written was awkward. The authors put the introduction, results, conclusion and discussion together in the text of their MS such that a very large portion of the MS was to describe previously published results related to their

results, leaving very limited spaces to describe, interpret and summarize their own results, given the large amounts of data, results and information from their analyses. This makes it difficult for readers to find what are the key and novel discoveries from their research.
Thanks for your suggestions! The original format was in a letter format. We have converted into an article format. We have also improved our manuscript to focus on our findings.

The reviewer’s remarks are in black. Author’s reply is in blue.

Reviewer #4 (Remarks to the Author):

General reply:

We revised the manuscript, upon suggestions from the editor and reviewers. The major changes are:

1. It is in the Article format now. Previously it was in the Nature Genetics Letter format.
2. We did try to do digital pedigree studies as reviewer 1 suggested, but it turned out to be too difficult for the following reasons: 1: the pedigree information of many accessions was not well documented or is not available; 2: many accessions that were used to develop the accessions in this study have not been resequenced; 3: many accessions in this study are very close to each other in relationships, making it hard to determine the parent-child relationships. So instead, we performed the kinship analysis and think that our results revealed some important kinship information among these 1,143 indica accessions, such as 98 3R, 12 2R, and 3 CR accessions among 1,143 accessions in this study have relationship with a 0.45+ kinship coefficient.
3. We have performed the FPVD analyses on the simulated “inferior” 3- and 2-line hybrids (to mimic the unreleased hybrids), and have added the results to the manuscript. They can serve as controls for analyses of superior (released) hybrids.
4. Most figures are in zoomable high definition PDF format now, except the screenshots in the manuscript file in MS-Word

This study by Lv et al. aimed as they say in the abstract of their study L39 "... goals were to obtain complete genetic variation information for these accessions and to comprehensively identify the key gene loci for heterosis of hybrid rice...". They genome sequenced >1000 Indica parental lines that were used in hybrid varieties to answer this goal. However reading the manuscript I'm not sure which analysis leads to the insight on the loci responsible for heterosis.

In my mind I thought heterosis is simply the effect of heterozygous genotypes as they were formed from parents with different genotypes. The study they cite in the manuscript (Huang et al 2015 Nat comm) had basically done what the authors set out to answer as they indicate in the abstract. Here in this manuscript they simply look at allele frequency differences of the parents which the frequency difference of itself has really nothing to do with heterosis per se. There's no analysis that sets out to see if those parental alleles are responsible for heterosis.

What they chose to do is simply look at sites where the parents have very different allele frequencies. First I wasn't sure how the parents were grouped to calculate the allele frequency. In fact the M&M is so sparse that its hard to see the details of their method. For instance how do they deal with the fact that not all polymorphic sites will have the same allele count because of missing genotype calls naturally arising from next gene sequencing data? How much of the difference is due to simply the underlying population structure and hence has no heterosis effect when brought together. No direct connection between heterosis sites and parental genotypes makes this study not what it set out to do in its title and abstract.

Also there are some contents of the manuscript where as a reader wonders why its there. For instance the whole half of this manuscript deals with explaining the genetic relationships of the parents. This is a pretty short study but half just simply explaining who's related to who seems a waste of a space especially when its not really that relevent to their main topic.
The Cd tolerance, PAV analysis, and GWAS results are also not so relevent to the manuscript as they really have nothing to do with heterosis either. Why were they there for?
Lastly the result they present are essentially for the most validating previous studies. The whole study reads like a short validation study. The dataset they generate is great but the analysis doesn't seem to be on par with what they set out to do. In fact this type of manuscripts frankly I've read more frequently in a more specialized journal like Rice.

Thanks for reading our manuscript and providing the important feedbacks! The rationale behind FPVD analyses in this study is that among superior hybrid parents, the most shared parental genetic differences are most relevant to the heterosis. The newly added FPVD analyses on the simulated “inferior” 3- and 2-line hybrids did show the sterility-related the genes are in the loci with high FPVD values. Therefore, FPVD results on the superior hybrids should be credible. As far as we know, no one has used the FPVD approach before, thus it is a new way to identify the heterosis genes and loci.

Huang et al (2015) found some heterosis related loci, and in 2016 they suggested that the yield heterosis in 3- and 2-line hybrid rice is controlled by very few but different genes, namely, *hd3a* and *tac1* in 3-line, *LAX1* and *Ghd8* in 2-line, based on studies on 15 *indica* hybrids. Our studies on 415 3-line superior hybrid, 136 2-line superior hybrid showed many more loci and genes are probably related to the heterosis. In addition, our results are based on the higher coverage depth NGS data, with a better reference genome, another reason that our results should be better than Huang’s two studies in 2015 and 2016, although we cannot eliminated the possibility that some related variation information was missing, either due to missing genotype calls naturally arising from next gene sequencing data, or being removed during filtering.

Theoretically some parental genetic variation differences could be simply the underlying population structure and thus not related heterosis. If there is any of these, we assume that these false positives would have stood out in our FPVD analysis on the simulated “inferior” hybrids. However, the highest peaks of FPVD analysis on the simulated “inferior” hybrids are the sterility-related loci. Therefore the false positives seem not a problem in our study.

We included the Cd and PAV studies because it is an example of values of the rich genetic information our NGS resequencing data provide.

Here are some minor changes: we improved our manuscript to focus on our findings; modified the title, to make it fit the content better; since there is no new findings, we have removed GWAS parts.

Again, thank you very much for your valuable feedbacks!

REVIEWERS' COMMENTS:

Reviewer #1 (Remarks to the Author):

Most of my concerns have been addressed. However, there are two remaining problems:
1. The authors stated that "we have now changed to the article format with a formal abstract". However, the abstract of this version is still unsatisfactory, and needs to be reorganized. The writing of the mani text also needs to be polished.
2. Kinship analysis, phylogenetic trees and population structure analysis are actually duplicated analyses, without new informatin provided. I suggest the authors remove Table 1 and Fig. 1.

Reviewer #3 (Remarks to the Author):

This version of the MS has been much improved

[Editor: Reviewer #4 states in Remark to Editor section that there is limited new biology associated with this resource study and leaves the decision to editors.]

Reviewer #1 (Remarks to the Author):

Our replies are in blue text.

Most of my concerns have been addressed. However, there are two remaining problems:

1. The authors stated that "we have now changed to the article format with a formal abstract". However, the abstract of this version is still unsatisfactory, and needs to be reorganized. The writing of the main text also needs to be polished.

   Thank you for letting us know. We have modified the Abstract and Introduction of the main text, and we have enlisted help from Nature Spring English Editing Service to polish the language. We hope that the manuscript has been improved.

2. Kinship analysis, phylogenetic trees and population structure analysis are actually duplicated analyses, without new information provided. I suggest the authors remove Table 1 and Fig. 1.

   Thank you for your suggestion. We have accepted your suggestion about Table 1 and moved it to the Supplementary section as Supplementary Table 1.

   We attempted to carry out some digital pedigree analysis upon a suggestion from a reviewer after the initial peer review, but we failed due to the reasons stated in the “Phylogenetic and kinship study” result section of the revised manuscript. Therefore, we performed kinship analysis instead. The results did reveal the very close relationships between some accessions that will likely be very interesting to many rice genetic researchers and breeders, and they may be better than the results of the phylogenetic study in this regard. In addition, the small phylogenetic trees in Fig. 2 b,c help to illustrate the hybrid cross patterns but are not the main focus of Fig. 2. We would prefer to retain Fig. 1 in the main text. Thanks for your understanding in advance!
